# Loss of MLKL impairs abdominal aortic aneurysm development by attenuating smooth muscle cell necroptosis

**DOI:** 10.1038/s41419-026-08427-4

**Published:** 2026-02-11

**Authors:** Harshal Nemade, Dennis Mehrkens, Hannah Sophia Lottermoser, Zeynep Ece Yilmaz, Julian Mader, Patrik Schelemei, Felix Ruben Picard, Simon Geißen, Gülsah Fülgen Schwab, Friedrich Felix Hoyer, Henning Guthoff, Alexander Hof, Felix Sebastian Nettersheim, Agapios Sachinidis, Norbert Gerdes, Gerhard Sengle, Holger Winkels, Stefan Baldus, Manolis Pasparakis, Martin Mollenhauer, Matti Adam

**Affiliations:** 1 Department for Experimental Cardiology, Faculty of Medicine, University of Cologne, and Clinic III for Internal Medicine, University Hospital Cologne, Cologne, Germany; 2 Center for Molecular Medicine Cologne (CMMC), Faculty of Medicine and Faculty of Mathematics and Natural Sciences, University of Cologne, Cologne, Germany; 3 Division of Cardiology, Pulmonology and Vascular Medicine, Medical Faculty and University Hospital, Heinrich-Heine University, Düsseldorf, Germany; 4 Cardiovascular Research Institute Düsseldorf (CARID), Medical Faculty and University Hospital, Heinrich-Heine University, Düsseldorf, Germany; 5 Department of Pediatrics and Adolescent Medicine, Faculty of Medicine and University Hospital Cologne, University of Cologne, Cologne, Germany; 6 Center for Biochemistry, Faculty of Medicine and University Hospital Cologne, University of Cologne, Cologne, Germany; 7 Cologne Center for Musculoskeletal Biomechanics (CCMB), Cologne, Germany; 8 Cologne Excellence Cluster on Cellular Stress Responses in Aging-Associated Diseases (CECAD), University of Cologne, Cologne, Germany

**Keywords:** Necroptosis, Aortic diseases

## Abstract

Abdominal aortic aneurysm (AAA) is a life-threatening condition characterized by chronic vascular inflammation and progressive aortic wall deterioration. MLKL-driven necroptosis, a highly inflammatory form of cell death, has been implicated in several cardiovascular pathologies; however, its role in AAA remains incompletely understood. Using the aortic elastase-perfusion model, we investigated the impact of necroptosis deficiency on AAA progression in necroptosis-deficient transgenic mice, including RIPK1 kinase-inactive (*Ripk1*^*D138N/D138N*^), MLKL knockout (*Mlkl*^*−/−*^), and MLKL phospho-deficient (*Mlkl*^*AA*^) animals. Ultrasound analysis revealed that, compared to WT animals, the necroptosis-deficient animals were protected from aneurysm formation, exhibiting preserved aortic structure, reduced immune cell infiltration, and attenuated extracellular matrix remodeling. Bulk mRNAseq revealed significant downregulation of genes associated with fibrinolysis, immune cell activation/migration, inflammation, complement and coagulation cascades in necroptosis-deficient animals. Bone marrow transplantation experiments demonstrated that MLKL deficiency in smooth muscle cells (SMCs), rather than in myeloid cells, was primarily responsible for the protective phenotype. Furthermore, consistent with previous reports, necroptosis induction in MLKL-expressing human and primary mouse SMCs led to increased secretion of proinflammatory cytokines. Live-cell imaging revealed that necroptotic SMCs promote activation and migration of HL60-differentiated polymorphonuclear neutrophils. Collectively, these findings demonstrate that necroptotic SMC death and resulting leukocyte activation play a causative role in AAA development and suggest that pharmacological inhibition of MLKL may represent a promising treatment strategy for AAA disease.

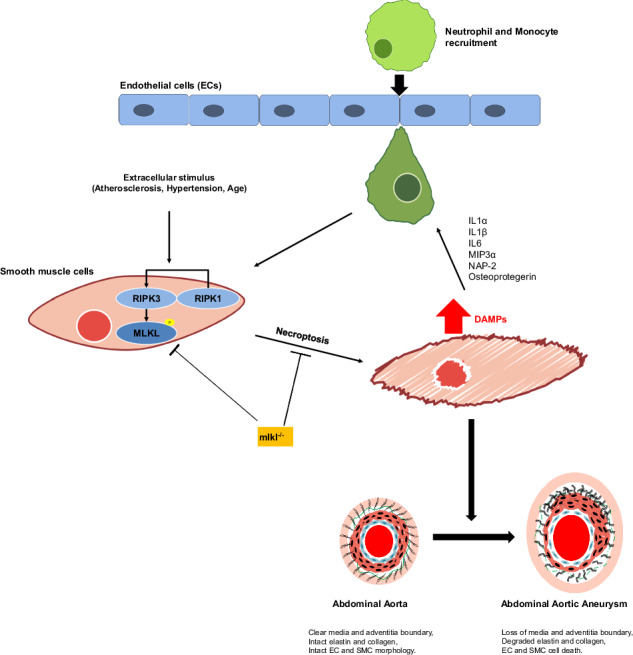

## Introduction

Abdominal aortic aneurysm (AAA) formation is a common pathology in Western countries with an incidence of around 2.5% [1]. AAA disease is associated with aortic complications, including rupture or dissection, which occur in 5% of all patients and have a poor prognosis [2]. Therefore development of therapeutic options to inhibit AAA formation and progression is of high clinical importance. Loss of smooth muscle cells (SMC) in the tunica media of the aortic wall represents a central event in the development of AAA, and apoptotic cell death has been early identified as a critical mechanism of SMC degradation in this disease [3]. Apoptosis is a well-characterized form of regulated cell death orchestrated by a family of cysteine proteases known as caspases [4]. Apoptosis was considered for many years the only type of regulated cell death. However, the recent discovery of molecularly controlled pathways of lytic cell death, such as necroptosis, revealed that also necrotic cell death, which was initially suggested to represent passive, uncontrolled and “accidental”, can be regulated [5]. Recent studies identified necroptosis as a regulated necrotic cell death process that is induced by receptor-interacting kinases 3 (RIPK3) and its substrate, mixed lineage kinase-like protein (MLKL) [6]. Necroptosis is triggered by various stimuli such as toll-like receptor (TLR) activation, interferon-gamma (IFN-γ), or intracellular DNA and RNA recognition, with tumor necrosis factor alpha (TNF-α) signaling being the most studied mechanism until now. Activation of the TNF-α receptor results in trans- and autophosphorylation of RIPK1, which interacts with RIPK3 to induce the assembly of the necrosome complex that facilitates the subsequent phosphorylation of MLKL by RIPK3 at serine S345 and S347 [7, 8]. MLKL phosphorylation exposes its N-terminal domain, inducing trimerization and translocation of MLKL to plasma membrane thereby “executing” necroptotic cell death by inducing Ca^2+^ influx and cellular lysis [9]. In contrast to apoptosis, which is considered a non-inflammatory type of cell death, necroptosis serves as a potent inducer of inflammation and has been linked to inflammatory diseases of the skin and the gut, as well as to chronic liver inflammation, neurodegenerative pathologies and cancer [10].

The formation and progression of AAA disease is a highly inflammatory process. Progressive dilation of the aorta is associated with the recruitment and activation of leukocytes, such as polymorphonuclear neutrophils (PMN) and macrophages. Subsequent inflammatory activation of myeloid cells leads to the production of reactive oxygen species (ROS) and pro-inflammatory cytokines, causing subsequent inflammatory cell infiltration. These processes finally elicit degradation of the extracellular matrix (ECM) by matrix metalloprotease (MMP) activation, which further drives AAA progression. Necroptosis-induced membrane permeabilization rapidly releases cellular components and damage-associated molecular patterns (DAMPs) [11], which are recognized by resident aortic macrophages. In turn, this leads to the formation of the NLRP3 inflammasome, triggering further production of proinflammatory cytokines and chemokines, including TNF-α and IL-1β, a hallmark of AAA development [12, 13].

Although depletion of PMN [14] or IL-1β [15] inhibition showed promising results in animal models and clinical trials have confirmed the efficacy of anti-inflammatory therapies in atherosclerotic cardiovascular diseases (CANTOS[16], COLCOT [17]), no targeted therapies for AAA disease are available so far [18]. Pharmacological inhibition of RIPK1 and genetic loss of RIPK3 ameliorate AAA progression in mice, pointing to cell death as a potential target in AAA disease treatment [19, 20]. Nonetheless, given the dual role of both kinases being involved in necroptosis and in apoptosis, the specific role of necroptotic cell death in AAA remains unclear. Whether necroptotic stimuli in the early phase of AAA formation activate leukocytes, and/or the recruitment of leukocytes drives the extent of aneurysm formation by induction of additional necroptosis, remains to be elucidated.

## Results

### Loss of MLKL attenuates aneurysm formation in mice

**Fig. 1:**
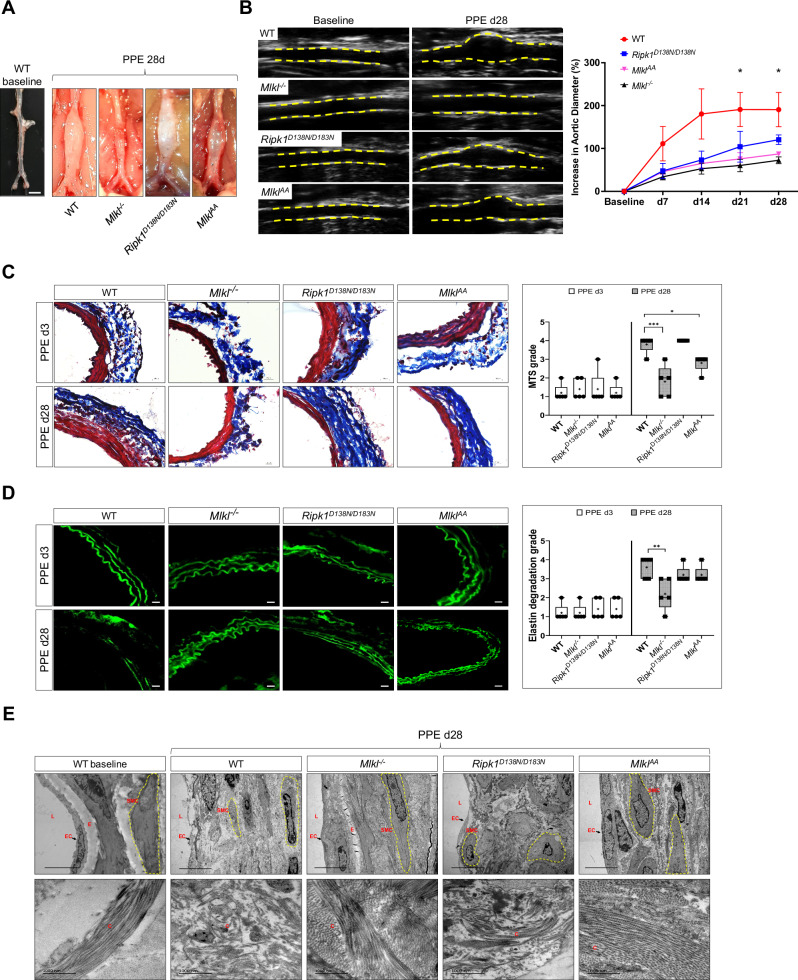
Necroptotic cell death is crucial for aortic dilation and vascular remodeling in AAA. **A** Representative macrograph of abdominal aorta at baseline and 28 days post-PPE (scale bar = 1 mm). **B** Representative B-mode ultrasound images of abdominal aorta at baseline and 28 days post-PPE. Yellow lines indicate vascular wall. Assessment of aortic diameter represented as percent increase to baseline diameter (*n* = 10). **C** Representative images of abdominal aortic sections stained with Masson’s Trichrome Staining (MTS) (scale bars = 20 μm) and quantification of aortic collagen content (red) by MTS grade analyses (*n* = 5). **D** Representative images of elastin autofluorescence of abdominal aortic sections (green, scale bars = 20 μm) and quantification of elastin strand breaks by elastin degradation grade analysis (*n* = 5). **E** Representative transmission electron microscopy (TEM) images of abdominal aortic sections at baseline and 28 days post-PPE displaying smooth muscle cells (SMC) morphology, endothelial cell (EC) abundance and elastin (E) degradation (upper panel) and collagen (C) structure and fiber arrangement (lower panel); (L = Lumen; scale bars = 1000 nm). Data are expressed as mean ± SD. Statistical significance was determined by one-way ANOVA with Tukey’s multiple comparisons test for. * = *P* < *0.05*, ** = *P* < *0.01*, *** = *P* < *0.005*, **** = *P* < *0.001*).

To investigate the role of MLKL induced-necroptosis in the development and progression of AAA disease, 10- to 14-week-old male WT, Mlkl knockout (*Mlkl*^*–/–*^), RIPK1 kinase-inactive (*Ripk1*^*D138N/D138N*^) and MLKL phospho-mutated (*Mlkl*^*AA*^) mice were subjected to PPE surgery. *Mlkl*^*–/–*^, *Mlkl*^*AA*^ and in part, *Ripk1*^*D138N/D138N*^ animals showed an attenuation of aortic diameter increase as assessed by an ultrasound analysis, whereas ~70% of WT animals developed PPE-induced AAA (Fig. 1A, B and Supplementary Fig. S1A). Masson’s trichrome staining (MTS) and elastin fluorescence imaging showed that aortic structural alterations, collagen degradation and elastin disarray were ameliorated in *Mlkl*^*–/–*^ and *Mlkl*^*AA*^ animals; however, less significant improvements in these aortic wall structural components were observed in *Ripk1*^*D138N/D138N*^ mice 3 and 28 days post-PPE (Fig. 1C, D and Supplementary Fig. S1B–D). Grading criteria for MTS and elastin images are outlined in Supplementary Fig. S2A, B). TEM micrographs demonstrated decreased EC and SMC cell volume, loss of SMC membrane integrity and disarranged collagen fibers in WT animals subjected to AAA. These alterations were strongly attenuated in *Mlkl*^*–/–*^, *Mlkl*^*AA*^ and, in part, in *Ripk1*^*D138N/D138N*^ animals (Fig. 1E upper panel). Furthermore, WT animals developed disarranged and fragmented collagen fiber structure post-PPE, which was not detectable in *Mlkl*^*–/–*^ and *Mlkl*^*AA*^ mice (Figs. 1E lower panel and Supplementary Fig. S2C). Interestingly, *Ripk1*^*D138N/D138N*^ animals showed marked changes in SMC morphology, elastin degradation and loss of ECs as compared to WT.

### Loss of MLKL abrogates the infiltration of immune cells in PPE-induced AAA lesions

**Fig. 2:**
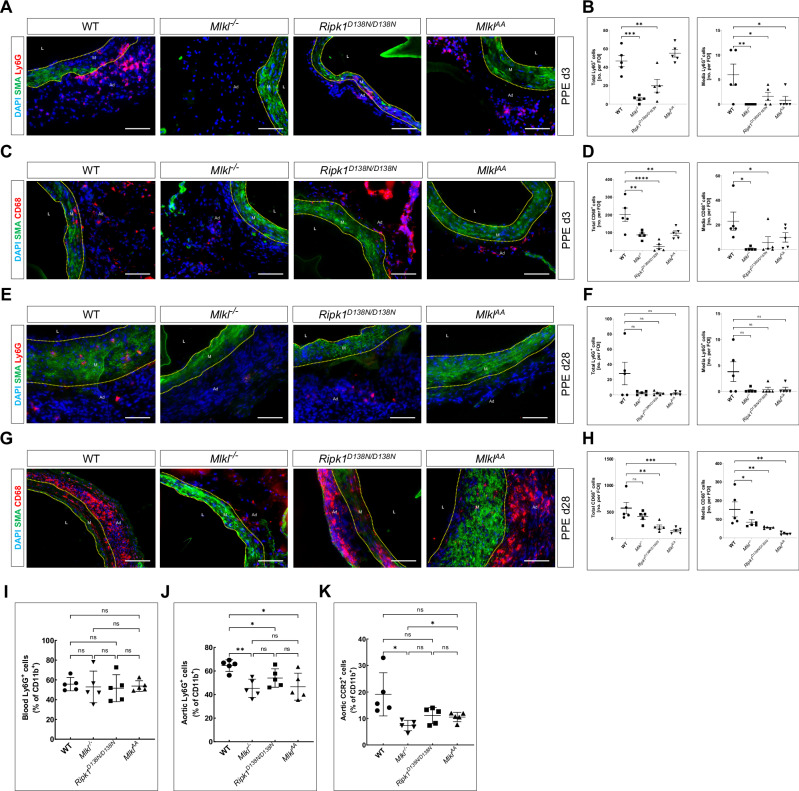
Lack of MLKL reduces proinflammatory immune cell infiltration during the early stages of AAA. **A**, **B** Representative immunofluorescence images and quantification of Ly6G⁺ neutrophils (red) and **C**, **D** CD68⁺ macrophages (red) within aneurysmal aortic segments 3 days post-PPE; graphs show total cell counts (left) and medial cell counts (right). **E**, **F** Representative immunofluorescence images and quantification of Ly6G⁺ neutrophils (red) and **G**, **H** CD68⁺ macrophages (red) within aneurysmal aortic segments 28 days post-PPE; graphs show total cell counts (left) and medial cell counts (right). Scale bar = 20 μm; vascular lumen (L), tunica media (M), tunica adventitia (Ad). Statistical significance was determined by Ordinary one-way ANOVA with Holm-Šídák multiple correction test; * = *P* < *0.05*, ** = *P* < *0.01*, *** = *P* < *0.005*. Flow cytometric analysis of total Ly6G^+^ neutrophils in the blood (**I**) and abdominal aortic tissue (**J**), and of CCR2^+^ cells in the abdominal aortic tissue (**K**), from PPE-operated animals at 3 days post-PPE (*n* = 5). All data are expressed as mean ± SD. Statistical significance was determined by Brown-Forsythe and Welch ANOVA tests with Welch’s correction; * = *P* < *0.05*, ** = *P* < *0.01*, *** = *P* < *0.005*.

To determine whether necroptosis deficiency abrogates the infiltration of immune cells, the abdominal aorta of mice was harvested and analyzed by immunofluorescence staining, histology and flow cytometry. At baseline conditions, no Ly6G^+^ PMN or CD68^+^ macrophages could be detected within the aortae of WT animals (Supplementary Fig. S3A). At 3 days post-PPE, WT animals exhibited marked infiltration of Ly6G^+^ neutrophils within AAA lesions, whereas *Mlkl*^*–/–*^, *Ripk1*^*D138N/D138N*^ and *Mlkl*^*AA*^ mice harbored significantly fewer Ly6G^+^ cells in the tunica media (Fig. 2A, B and Supplementary Fig. S3B). Similarly, CD68^+^ macrophage numbers were increased in the aortic media of WT animals but were attenuated in *Mlkl*^*–/–*^, *Ripk1*^*D138N/D138N*^ and *Mlkl*^*AA*^ mice (Fig. 2C, D and Supplementary Fig. S3C). Interestingly, the location of neutrophils and macrophages coincided with the loss of alpha smooth muscle actin (aSMA) signal within the tunica media, indicating enhanced SMC loss at the site of inflammatory cell infiltration. To investigate the effects of necroptosis deficiency on long-term leukocyte infiltration, we harvested aortas from all genotypes 28 days post-PPE. At this time point, Ly6G^+^ neutrophils were barely detectable (Fig. 2E, F and Supplementary Fig. S3D), whereas CD68^+^ macrophage infiltration in the tunica media was significantly attenuated across all three genotypes (Fig. 2G, H and Supplementary Fig. S3E).

To further investigate the effect of MLKL in aortic inflammation during AAA development, we performed flow cytometry analyses of the aorta and blood of mice 3 days post-PPE. The detailed gating strategy is shown in Supplementary Fig. S3F, G. Although total PMN blood count was unaffected, total aortic PMN infiltration was significantly attenuated in all three necroptosis-deficient animals with most significant reduction observed in the *Mlkl*^*–/–*^ animals compared to WT animals (Fig. 2I, J). We also observed a reduction in CCR2^+^ macrophage numbers in *Mlkl*^*–/–*^ animals (Fig. 2K). Combined, this tissue and FACS analysis showed that necroptosis deficiency selectively attenuates neutrophil and macrophage infiltration into the aneurysmal wall without affecting the circulating leukocyte levels, thereby preserving SMC integrity and reducing inflammatory injury during AAA development.

### Necroptosis deficiency downregulates inflammation and fibrinolysis-related genes in AAA

**Fig. 3:**
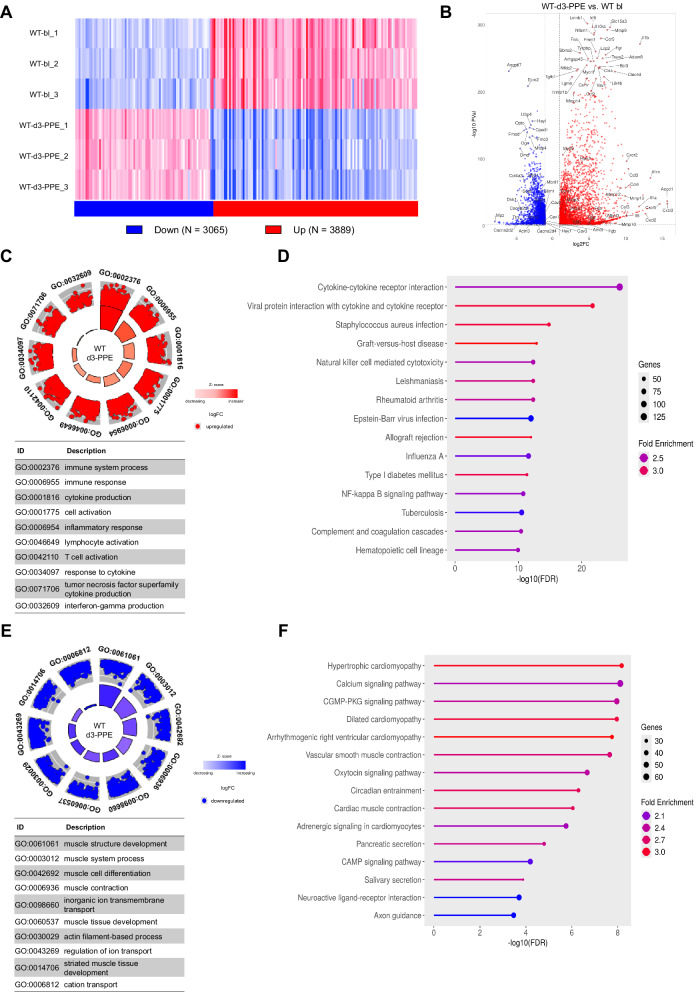
AAA aortae acquire an inflammatory transcriptional phenotype. Bulk transcriptome analysis of aortic tissues from WT animals harvested at baseline (WT-bl) and 3 days post-PPE (WT-d3-PPE) (*n* = 3). **A** Heatmap visualizing the significantly deregulated differentially expressed genes (DEGs) and **B** Volcano plot highlighting significantly upregulated (red) and downregulated (blue) DEGs; top 100 DEGs are labeled. GOcircle plot (**C**) and KEGG pathway enrichment analysis (**D**) for upregulated genes. The GOcircle plot displays log fold change (logFC) alongside the top 10 enriched GO terms (table), with inner bars indicating significance (–log10 adjusted *P*-value) and color representing enrichment Z-score (GOplot, R). KEGG pathways are shown as dot plots, where dot size indicates gene count per pathway and color indicates fold enrichment (SRplot). GOcircle plot (**E**) and KEGG pathway enrichment analysis (**F**) for downregulated genes presented as in (**C**, **D**).

Abdominal aortic tissue samples were collected from WT animals 3 days post-PPE (WT-d3-PPE), and bulk mRNAseq was performed. Unoperated WT animals were used as baseline control (WT-bl). The mRNAseq analysis identified 6954 differently expressed genes (DEGs) (≥ 1 log2FC, *p* > 0.05) in WT-d3-PPE animals, consisting of 3889 upregulated and 3065 downregulated genes (Fig. 3A, B). A majority of the upregulated genes were enriched in cytokine production, immune and inflammation response pathways, leukocyte-, neutrophil- and macrophage-migration related GO (Gene Ontology) terms, comprising for example *Cxcl3* (log2FC = 15.65), *Cxcl5* (log2FC = 14.26), *Il1a* (log2FC = 12.12), *Il1b* (log2FC = 11.78), *Ccl3* (log2FC = 10.69), *Il6* (log2FC = 10.55), and *Csf3* (log2FC = 10.25) (Fig. 3C, D and Supplementary Fig. S4A) whereas most downregulated genes were enriched in muscle contraction, smooth muscle cell contraction and cation transport related GO terms for example *Myh6* (log2FC = –6.85), *Slc2a5* (log2FC = –6.04), *Mstn* (log2FC = –5.88), *Mb* (log2FC = –5.25), *Myl1* (log2FC = –4.98), *Myh6* (log2FC = –6.85), and *Cacna1s* (log2FC = –3.04) (Fig. 3E, F and Supplementary Fig. S4B). These findings correlate with our observed increase in aortic Ly6G^+^ and CD68^+^ cells. In addition to inflammation-related processes, genes involved in fibrinolysis, including *Hrg*, *Fga*, *Fgg*, *F2*, *Fgb*, *Apoh* and *Plg* were significantly upregulated in WT-d3-PPE animals (Supplementary Fig. S4C).

**Fig. 4:**
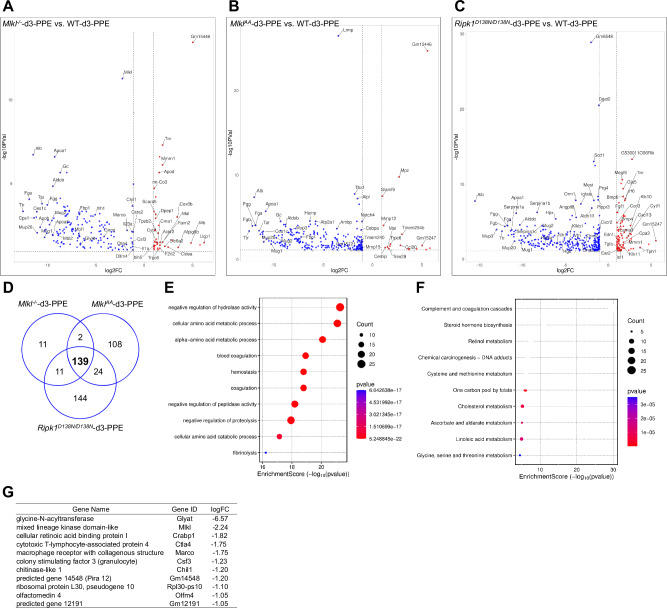
Necroptosis deficiency downregulates inflammation- and fibrinolysis-related genes in AAA. Volcano plots showing significantly upregulated (red) and downregulated (blue) DEGs in *Mlkl*^*–/–*^-d3-PPE (**A**), *Mlkl*^*AA*^-d3-PPE (**B**) and *Ripk1*^*D138N/D138N*^-d3-PPE (**C**) mice compared to WT-d3-PPE mice. **D** Venn diagram showing the overlap of downregulated genes among *Mlkl*^*–/–*^, *Mlkl*^*AA*^ and *Ripk1*^*D138N/D138N*^ mice at 3 days post-PPE. GO term bubble plot (**E**) and KEGG pathway enrichment analysis (**F**) displaying top enriched biological processes and pathways respectively from commonly downregulated 139 genes between all three genotype. The bubble size representing gene count and color indicating enrichment score (–log10 (*p*-value)). **G** List of 11 uniquely downregulated genes in *Mlkl*^*–/–*^-d3-PPE animals with their respective logFC values.

Next, we performed DEG analysis between WT-d3-PPE and the necroptosis-deficient animals. Our data showed 39 up- and 163 downregulated genes in *Mlkl*^*–/–*^-d3-PPE animals, 20 up- and 274 downregulated genes in *Mlkl*^*AA*^-d3-PPE animals, and 93 up- and 318 downregulated genes in *Ripk1*^*D138N/D138N*^-d3-PPE animals (Fig. 4A–C and Supplementary Fig. S4D–S4F). Interestingly, we identified 139 commonly downregulated genes between necroptosis-deficient animals (Fig. 4D). As shown in Fig. 4E, the biological process (BP) enrichment analysis showed that these 139 downregulated genes were significantly involved in negative regulation of hydrolase activity, cellular and alpha-amino acid metabolic processes, blood coagulation, and fibrinolysis. KEGG pathway analysis showed that these downregulated genes were mainly enriched in metabolic pathways like Complement and coagulation cascade, Retinol metabolism, Cysteine and methionine metabolism, Cholesterol metabolism, Linoleic acid metabolism and Glycine, serine and threonine metabolism (Fig. 4F and Supplementary Fig. S5A–D). In addition, we also identified 11 uniquely downregulated genes in *Mlkl*^*–/–*^-d3-PPE animals as compared to *Mlkl*^*AA*^-d3-PPE and *Ripk1*^*D138N/D138N*^-d3-PPE animals, which included known AAA and inflammation-associated genes like *Ctla4* [21], *Marco* [22, 23], *Csf3* [24], *Chil1* [25] and *Olfm4* [26, 27] (Fig. 4G). Downregulation in these genes further supports our initial observations of significantly reduced inflammatory cell infiltration in aortic lesions of *Mlkl*^*–/–*^ animals post-PPE. KEGG pathway analysis of these 11 genes showed enrichment in IL-17 signaling pathway and TNF signaling pathway (Supplementary Fig. S6A). We also performed similar GO and KEGG pathway analysis for uniquely downregulated genes in *Mlkl*^*AA*^-d3-PPE and *Ripk1*^*D138N/D138N*^-d3-PPE animals. The 108 downregulated genes in *Mlkl*^*AA*^-d3-PPE animals were mainly involved in musculoskeletal movement, muscle contraction, muscle development and pathways like Hypertrophic cardiomyopathy, Dilated cardiomyopathy, Tryptophan metabolism etc.; on the other hand, the 144 downregulated genes in *Ripk1*^*D138N/D138N*^-d3-PPE animals were mainly involved in steroid metabolic process, lipid homeostasis, triglyceride metabolic process, fatty acid metabolic process and pathways like Cholesterol metabolism, Insulin resistance, Pyrimidine metabolism and Glutathione metabolism etc. (Supplementary Fig. S6A). List of common genes between 2 groups is shown in Supplementary Fig. S6B.

Our mRNAseq data showed only one commonly upregulated gene, Mal (log2FC = 1.5 ± 2), between necroptosis-deficient animals. Analysis of uniquely upregulated genes revealed an enrichment in pathways related to reactive oxygen species and wound healing in *Mlkl*^*–/–*^-d3-PPE animals. Whereas, in *Mlkl*^*AA*^-d3-PPE and *Ripk1*^*D138N/D138N*^-d3-PPE animals, an enrichment in interleukin-1 production, leukocyte chemotaxis, IL-17 signaling pathway, and epithelial cell proliferation, PI3K-Akt signaling pathway, Cytokine-cytokine receptor interaction and Ras signaling pathway was observed (Supplementary Fig. S7A). List of common upregulated genes between the groups is shown in Supplementary Fig. S7B.

Platelet activation and aggregation pathways are known to play a crucial role in AAA progression [28]. Platelet-related genes like *Selp* (P-selectin), *Sell* (L-selectin), and *Pf4* were upregulated in all day-3 PPE aortas irrespective of the genotype, whereas the expression of several other genes (*Alox12, GP5, Treml1, Mpig6b, MPL, Gp1ba, CD36, GP9, Itga2b*) remained unchanged (Supplementary Fig. S8A). Next, given the critical role of serine protease activity in ECM degradation, we investigated the expression of genes belonging to the serpin family, a group of serine protease inhibitors that regulate proteolysis, tissue remodeling and inflammatory processes. In WT aortas 3 days post-PPE, we observed robust upregulation of *Serpina1a*, *Serpina1b*, *Serpina1c*, *Serpina1d*, *Serpina1e*, *Serpina6*, and *Serpina11* relative to baseline. In contrast, these transcripts remained largely unchanged in all three necroptosis-deficient genotypes compared to WT-bl and were significantly downregulated relative to WT-d3-PPE. Interestingly, three members of *Serpina3* subfamily (*Serpina3f*, *Serpina3g* and *Serpina3n)* were consistently upregulated across all genotypes, with *Serpina3n* showing the highest expression in *Mlkl*^*–/–*^-d3-PPE animals (Supplementary Fig. S9A).

In summary, necroptosis deficiency seems to dampen the complement cascade, retinol metabolism, inflammatory-, fibrinolytic- and coagulation-related responses in AAA. Although some genes involved in the platelet pathway were upregulated after PPE, they were not altered by necroptosis deficiency, indicating that AAA protection might be primarily mediated through reduced SMC necroptosis and inflammation.

### Lack of MLKL in abdominal aortic SMCs is protective against AAA development

**Fig. 5:**
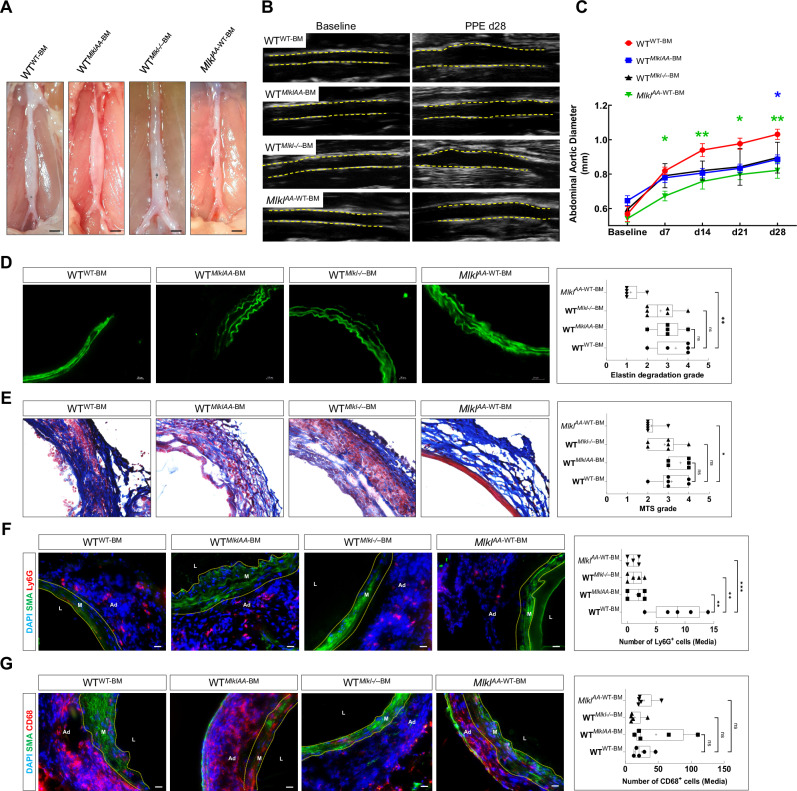
MLKL deficiency in aortic SMC is protective against AAA development. **A** Representative macrographs of abdominal aortas 28 days post-PPE (scale bar = 1 mm). B-mode ultrasound images at baseline and 28 days post-PPE, (yellow lines = vessel wall) (**B**) with quantification of aortic diameter (**C**) (*n* = 5). **D** Representative elastin autofluorescence images (green) and quantification of elastin degradation grade. **E** Representative Masson’s Trichrome-stained sections with qualitative assessment of aortic wall structures using MTS grading system (*n* = 5; scale bar = 20 μm). Representative confocal images showing α-SMA (green) with Ly6G⁺ neutrophils (red, **F**) or CD68⁺ macrophages (red, **G**), with quantification of number of cells observed infiltrated in aortic media. (*n* = 5; scale bar = 20 μm). L vascular lumen, M media, Ad adventitia. Data are mean ± SD; statistical significance by one-way ANOVA with Tukey’s test.

To investigate whether the loss of MLKL in vascular smooth muscle cells (vSMCs) or hematopoietic cells contributes to protection against AAA and reduced aortic leukocyte infiltration, we conducted a bone marrow (BM) transplantation study (Supplementary Fig. S10A). Irradiated wild-type (WT) recipient mice were reconstituted with BM from WT mice (WT^WT-BM^), *Mlkl*^*AA*^ mice (WT^MlklAA-BM^), or *Mlkl*^*–/–*^ mice (WT^Mlkl–/–-BM^). Additionally, irradiated *Mlkl*^*AA*^ recipient mice were reconstituted with WT BM (Mlkl^AA-WT-BM^). As expected, WT^WT-BM^ animals exhibited a significant increase in aortic diameter 28 days following the induction of AAA, whereas WT^MlklAA-BM^ and WT^Mlkl–/–-BM^ mice displayed mild increases in aortic diameter. Remarkably, Mlkl^AA-WT-BM^ animals were protected from AAA development (Fig. 5A–C). Histological analysis revealed that Mlkl^AA-WT-BM^ animals maintained stable aortic wall structures post-PPE. In contrast, other groups exhibited significant remodeling, including disorganized collagen, loss of medial layer boundaries and increased elastin fiber fragmentation (Fig. 5D, E and Supplementary Fig. S10B). We hypothesized that MLKL deficiency in SMCs prevents necroptosis, thereby reducing proinflammatory leukocyte infiltration in the Mlkl^AA-WT-BM^ aorta. Consistent with this hypothesis, Mlkl^AA-WT-BM^ animals 28 days post-PPE showed a significant reduction in Ly6G^+^ neutrophils in the abdominal aorta (Fig. 5F and Supplementary Fig. S10C, upper panel), although no significant change in CD68^+^ macrophage numbers was observed (Fig. 5G and Supplementary Fig. S10C, lower panel). Thus, MLKL deficiency in vSMCs appears to preferentially impair early neutrophil recruitment, while macrophage infiltration remains largely unaffected. All together, these findings suggest that loss of MLKL function in vSMCs provides protection against PPE-induced aneurysm formation, whereas its loss in hematopoietic cells confers only partial protection.

### MLKL-induced SMC death leads to release of DAMPs and promotes PMN activation

**Fig. 6:**
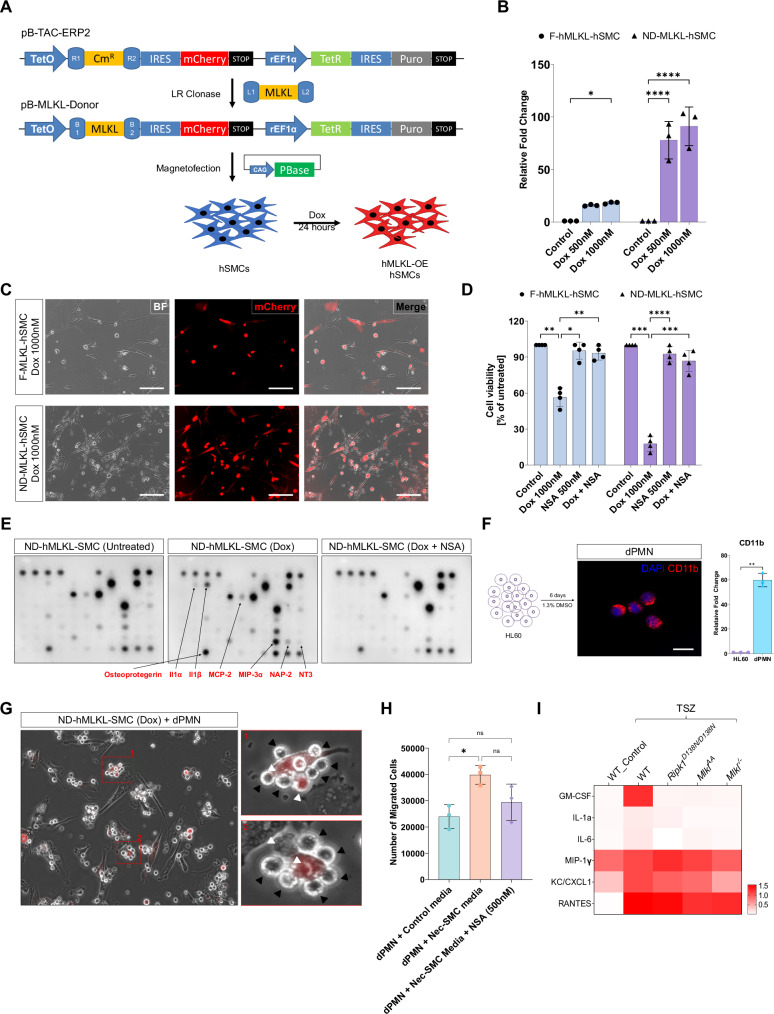
SMC necroptosis induces proinflammatory cytokine secretion and subsequent dPMN activation. **A** Cloning strategy for generating doxycycline-inducible full-length hMLKL (F-hMLKL) or “N”-domain of hMLKL (ND-hMLKL) expressing human SMCs. **B** qRT-PCR mRNA expression analysis of hMLKL before and 48 h after Doxycycline (Dox) induction (*n* = 3). **C** Representative images of mCherry fluorescence in 48 h Dox-induced F-hMLKL- and ND-hMLKL-hSMCs. **D** Cell viability assay of F-hMLKL- and ND-hMLKL-hSMCs under control, Dox, NSA, or Dox+NSA treatment conditions (24 h, *n* = 4). **E** Representative dot blots of secretome antibody array analysis of supernatants from ND-hMLKL-hSMCs treated with control, Dox, or Dox+NSA (24 h). **F** Differentiation protocol schematic to differentiate HL60 cells into polymorphonuclear neutrophil-like cells (dPMN), validated by CD11b staining and mRNA expression analysis (*n* = 3). **G** Co-culture images showing dPMN (black arrowheads) congregating around necroptotic hSMCs (white arrowheads). **H** Transwell migration assays were performed using dPMNs exposed to conditioned media from necrotic SMCs, in the presence or absence of an MLKL inhibitor, as the chemoattractant (*n* = 3). **I** Cytokine array analysis of supernatants from TSZ-treated primary mSMCs from WT, *Mlkl*^*−/−*^, *Mlkl*^*AA*^, and *Ripk1*^*D138N/D138N*^ animals (48 h, *n* = 3). All data are represented as mean ± SD; *P < 0.05, **P < 0.01, ***P < 0.005, **P < 0.001; Ordinary one-way ANOVA with Šidák’s multiple comparison test.

To show that MLKL-induced necroptotic SMC death is a crucial first step in the activation of PMN and subsequent AAA development, we generated two human aortic smooth muscle cell (hSMC) lines by transfecting Tet-On 3 G inducible expression constructs encoding for full-length human MLKL (F-hMLKL) and “N” domain of human MLKL (ND-hMLKL) (aa 1-182) (Fig. 6A) [29]. Doxycycline (Dox) induced robust production of the respective MLKL mRNA detected by qRT-PCR and proteins indirectly detected via the mCherry fluorescence signal (Fig. 6B, C). ND-hMLKL isoform was 4-fold higher expressed in comparison to the F-hMLKL isoform. This difference in expression was accompanied by significantly higher SMC death in ND-hMLKL-SMC (~80%) compared to F-hMLKL-SMC (~50%). Treatment of necrosulfonamide (NSA), a well-characterized hMLKL inhibitor, prevented SMC death in both cell lines (Fig. 6D). Live cell imaging of transfected and induced SMCs showed membrane rupture and cell lysis indicative of typical necroptotic death (Supplementary Video V1; yellow circles). Secretome antibody array analyses of MLKL expressing SMCs confirmed a significant increase in proinflammatory cytokine secretion, e.g., *Il1a*, *Il1b*, *Mcp2*, *Mip3a*, *Nap2*, *Nt3* and Osteoprotegerin, whereas necroptosis inhibition by NSA attenuated release of these cytokines (Fig. 6E and Supplementary Fig. S11A). Given that these cytokines play a substantial role in leukocyte recruitment in AAA disease[30], we generated polymorphonuclear neutrophil-like cells (dPMN) in vitro by differentiating HL60 cells and performed co-culture studies (Fig. 6F). Indeed, induction of necroptosis in SMCs rapidly triggered dPMN activation and migration, supporting the role of MLKL-mediated necroptosis as a driver of leukocyte recruitment and activation in AAA (Fig. 6G and Supplementary Video V2). The physiological relevance of these findings was confirmed in a transwell migration assay, showing that dPMNs were more attracted to necroptotic SMC- than to control supernatant, an effect partially blocked by the MLKL inhibitor NSA (Fig. 6H).

To determine whether the cytokine profile shifts from mouse SMCs (mSMCs) to those of human SMCs (hSMCs), we isolated mouse primary aortic SMCs and treated them with TNF-α, Smac mimetic, and zVAD (TSZ) to induce necroptosis. Secretome antibody array analysis revealed a significant increase in proinflammatory cytokine secretion in WT-SMCs, including GM-CSF, IL-6, MIP-1γ, and CXCL1. In contrast, necroptosis-deficient SMCs exhibited reduced cytokine release (Fig. 6I and Supplementary Fig. S12A).

## Discussion

We herein reveal that necroptotic cell death in SMCs is essentially involved in the pathogenesis of abdominal aortic dilation and potent mediator of vascular inflammation in AAA disease. We here provide the first evidence identifying the necroptotic executer protein MLKL as a major mediator of adverse vascular remodeling, leukocyte activation and infiltration and transcriptional alterations in AAA development. AAA is a multifactorial disease in which loss of vascular SMCs fundamentally contributes to aortic dysfunction and ECM degeneration, ultimately cumulating in fatal aortic complications such as rupture or dissection [31]. Furthermore, SMC-mediated paracrine effects on the tunica adventitia contribute to artery wall homeostasis with potent anti-inflammatory and anti-proteolytic properties [32]. Hence, prevention of SMC death is of primary importance in prevention of AAA growth and aortic rupture [33].

Given that cell death-inducing kinases, such as RIPK1, are not exclusively involved in the induction of necroptosis[34], we herein strive to reveal the role of necroptosis in AAA disease at different points of the necroptotic signaling cascade. To achieve this goal, we utilized three different mouse genotypes and subjected them to experimental AAA induction. MLKL-deficient animals (*Mlkl*^*–/–*^) lack the specific necroptosis execution protein and are considered fully “necroptosis” deficient. Accordingly, phosphorylation site mutated MLKL mice (*Mlkl*^*AA*^) [35] are not able to facilitate MLKL trimerization, translocation to and disruption of cellular membranes [35]. RIPK1 has been implicated both in apoptosis induction and, together with RIPK3, as an upstream mediator of necroptosis, whereas MLKL and its phosphorylated form act as the specific executioner of the necroptosis pathway [36]. AAA risk, aortic dilation, ECM degradation and SMC cell death were significantly decreased in all three mouse models, highlighting the prominent role of necroptotic processes in AAA. Interestingly, while *Mlkl*^*–/–*^ and *Mlkl*^*AA*^ mice showed high protection against AAA development, *Ripk1*^*D138N/D138N*^ mice were only partially protected from aortic dilation compared to WT mice, indicating that AAA formation is in part independent of RIPK1-induced apoptosis. TEM micrographs demonstrated decreased EC and SMC cell volume, loss of SMC membrane integrity and disarranged collagen fibers in the WT animals subjected to AAA. These alterations were strongly attenuated in *Mlkl*^*–/–*^, *Mlkl*^*AA*^ and, only in part, in *Ripk1*^*D138N/D138N*^ animals, which could explain partial AAA protection in this genotype. Furthermore, we detected upregulation in transcript levels of the serine protease inhibitor, *Serpina3n*, with most prominent expression in *Mlkl*^*–/–*^ animals. Interestingly, *Serpina3n* has already been reported to protect against aneurysm rupture by inhibiting granzyme B-mediated decorin cleavage, which may represent an additional protective mechanism [37].

Given the importance of inflammatory processes in AAA development, we investigated the role of necroptosis in aortic leukocyte infiltration. Necroptosis deficiency significantly reduced the number of Ly6G^+^ PMN and CD68^+^ macrophages within the tunica media and was associated with an improved number of SMCs compared to WT aneurysms, which showed significant loss of SMC population in tunica media identified by the loss of SMA signal. PMN activation is an important process in AAA development, and depletion of granulocytes has been shown to prevent AAA formation [14]. Nonetheless, our data presented here do not exclude a direct influence of necroptosis-derived, SMC-released DAMPs on monocyte/macrophage activation and infiltration, a process that is well established [38]. The question of which mechanism predominates remains an intriguing point for future studies.

Our bone marrow transplantation studies clearly indicated that loss of MLKL’s necroptosis-inducing function in SMCs prevents aortic diameter increase, stabilizes vascular wall integrity and significantly attenuates immune cell infiltration as compared to WT controls subjected to AAA. Mechanistically, these protective effects can be attributed to diminished SMC necroptosis which leads to reduction in the release of proinflammatory cytokines and DAMPs [11] via rapid permeabilization of the cell membranes as well as preventing release of inflammatory mediators activating pattern recognition receptors (PPRs) on neutrophils and macrophages [39]. Although these experiments indicate that necroptosis of SMCs is a major driver of AAA formation, additional effects of myeloid cell necroptosis could not be fully excluded since WT^MLKLAA-BM^ mice showed, at least in part, partial protection from aortic dilation.

We observed a selective reduction of neutrophils, but not macrophages, in Mlkl^AA-WT-BM^ aortas. This is consistent with studies showing neutrophil infiltration in the aortic wall peaks around day 3 after PPE surgery and declines thereafter [40]. At this early phase, these infiltrating neutrophils promote acute ECM degradation and aneurysm formation[41], whereas macrophages mainly sustain chronic ECM remodeling and inflammation and contribute to NETs degradation in later stages of the AAA pathology [42, 43]. Consistently, our in vitro data demonstrate that necroptotic SMC released pro-inflammatory cytokines and DAMPs cause robust PMN activation and migration [44], which can be attenuated by MLKL inhibition.

To gain deeper insights into the mechanistic role of necroptosis in aneurysm formation, we conducted bulk mRNAseq analysis of aortic tissue harvested 3 days post-PPE. In WT animals, consistent with previous reports, we observed significant downregulation of key genes associated with SMCs contractility, including *Acta2*, *Cacna2d1*, *Cnn1*, *Myh6*, *Myh11*, *Adamts6*, and *Mylk,* indicating loss of SMC contractility phenotype during AAA development. Overall, the downregulated genes were enriched in GO terms related to muscle contraction, actin filament-based process, cation transport, cytoskeleton organization, smooth muscle contraction, extracellular matrix assembly and aorta development processes critical for maintaining vascular wall structure and function[45–47]. Conversely, we observed robust upregulation of proinflammatory genes such as *Cxcl3*, *Cxcl5*, *Il1a*, *Il1b*, *Ccl3*, *Il6*, and *Csf3*, which correlate with immune cell infiltration and heightened vascular inflammation. These findings align with prior studies indicating that AAA pathogenesis involves pronounced recruitment of innate and adaptive immune cells, contributing to tissue degradation and remodeling[42, 48]. GO analysis further revealed enrichment of biological processes related to cytokine production, inflammatory responses, and the activation and migration of leukocytes, lymphocytes, neutrophils, and T cells; hallmarks of the inflammatory microenvironment observed in early AAA progression[48–50]. Together, our data support the notion that AAA formation involves a coordinated loss of SMC contractile function and an inflammatory milieu that promotes vascular wall degradation.

Interestingly, bulk mRNAseq data from necroptosis-deficient animals *Mlkl*^*–/–*^*, Mlkl*^*AA*^ and *Ripk1*^*D138N/D138N*^ showed significant suppression of genes associated with AAA pathogenesis. Notably, 139 genes were commonly downregulated across all three genotypes at 3 day post-PPE, hinting a conserved protective effect against early AAA development via suppression of necroptosis signaling. These genes are associated with biological processes like negative regulation of hydrolase activity, amino acid metabolism, blood coagulation, and fibrinolysis. Furthermore, KEGG pathway enrichment revealed that these genes are involved in metabolic and inflammatory pathways such as the complement and coagulation cascade, retinol and lipid metabolism, and amino acid metabolism, all of which are known to be upregulated in AAA[51–54]. Suppression of these pathways in necroptosis-deficient animals indicates a potential mechanistic link between necroptosis signaling and metabolic inflammation in aneurysm pathology.

Multiple studies, including human clinical investigations, have reported increased fibrinolytic activity in AAA, with elevated plasminogen-activating activity and fibrin turnover implicated in AAA development [55, 56]. In contrast, our mRNA-seq data show downregulation of multiple fibrinolysis- and coagulation-related genes in necroptosis-deficient aortas. Since intraluminal thrombi drive matrix degradation and SMC loss, reduced expression of fibrinolytic genes in necroptosis-deficient animals may provide an additional mechanism of protection against AAA. To our knowledge, this is the first report linking necroptosis deficiency to transcriptional suppression of fibrinolysis-associated genes in the context of AAA. Nonetheless, further studies at the protein and functional levels are required to determine whether these transcriptional changes translate into reduced fibrinolytic activity in vivo.

Platelet activation with subsequent intraluminal thrombus formation has been described as an important contributor to AAA by promoting leukocyte recruitment and ECM degradation [28]. In our study, mRNA-seq data demonstrated heterogeneous regulation of platelet activation markers in day-3 PPE aortas, with some transcripts being upregulated while others remained unchanged. These ambivalent results suggest that the upregulation of some platelet activation markers is unlikely to reflect altered platelet activation and aggregation, although definitive conclusions will require further investigation.

Importantly, genes such as *Marco,* involved in foam cell formation and matrix degradation[57, 58], *Csf3,* reported to be significantly increased in human AAA tissues[59, 60], *Chil1,* shown to be involved in vSMC phenotype switching [61, 62] and *Olfm4,* involved in pro-inflammatory response [63, 64] were uniquely downregulated in *Mlkl*^*–/–*^-d3-PPE animals. These findings corroborate our histological findings of reduced immune cell infiltration and further highlight the potential of anti-inflammatory benefits of necroptosis deficiency in AAA.

Finally, the relatively sparse set of commonly upregulated genes across the necroptosis-deficient models, with *Mal* being the only shared gene, further suggests that necroptosis deficiency exerts a predominantly suppressive effect on pro-inflammatory and metabolic transcripts. The unique upregulation of genes involved in oxidative stress responses and wound healing in *Mlkl*^*–/–*^-d3-PPE animals indicated towards reparative, rather than destructive, vascular environment.

Although our understanding of the underlying pathomechanism of AAA development and progression has greatly improved over the last decades, there is still no specific medical therapy available to slow the expansion rate of AAA [65] and surgical interventions such as open aneurysm repair (OAR) [66] or endovascular aneurysm repair (EVAR) [67] are still the gold standard by improving patient survival and health-related quality-of-life[68]. In this regard, cell death pathways, in particular apoptosis and necroptosis, have been studied extensively [69, 70]. This led to the discovery of potent antiapoptotic agents, such as Q-VD-OPh, 4-PBA, and L-NIL, which are currently under clinical investigation [71–74]. Necroptosis inhibitors targeting RIPK1 such as necrostatin-1 and necrostatin-1s, have already been shown to be effective against AAA development in mice models [20, 75, 76]. Nonetheless, RIPK1’s dual action in necroptosis and apoptosis regulation could explain observed off-target effects of these compounds thereby limiting their translation into a clinical approach[77]. Of note, several novel RIPK1 inhibitors like GSK’772 or GSK’547, are currently under investigation[78].

Supporting the translational relevance of our findings, analysis of publicly available transcriptomic datasets (GEO: GSM8328845 and GSM5557972) revealed a consistent trend toward increased MLKL expression in human AAA tissue (Supplementary Fig. S13A), in line with other studies reporting upregulation necroptosis signaling components in human aneurysms[19, 79]. In light of these data and the development of novel MLKL inhibitors currently under investigation, pharmacological MLKL inhibition may represent a promising therapeutic strategy for AAA prevention. Based on our in vitro study, specific inhibition of MLKL showed beneficial effects by reducing SMC death and dampening the secretion of proinflammatory cytokines. Although necroptosis-independent functions of MLKL cannot be fully excluded[80], targeting MLKL might be advantageous since it acts as the terminal executioner of necroptosis.

Taken together, this study not only dissects the critical role of MLKL-induced necroptosis in development of AAA but also identifies the therapeutic potential of targeting MLKL for prevention of AAA.

## Materials and methods

### Animal studies

Generation of *Ripk1*^*D138N/D138N*^ mouse line is described elsewhere[81]. The generation of *Mlkl*^*–/–*^ and *Mlkl*^*AA*^ strains will be described elsewhere[82]. All animal studies were approved by the local animal care authorities, Landesamt für Natur, Umwelt und Verbraucherschutz Nordrhein-Westfalen (LANUV), NRW, Germany (AZ: 2018.A030 and 2016.A212) and conformed to the guidelines from Directive 2010/63/EU of the European Parliament on the protection of animals used for scientific purposes. All experiments were performed on male mice, as AAA predominantly affects men and littermates were used as controls. All mice had free access to a standard laboratory diet (Altromin 1324 P Best, Altromin GmbH & Co. KG, Lage, Germany) and water. All surgical interventions were performed under isoflurane anesthesia and buprenorphine analgesia.

### PPE infusion model

To induce murine AAA, the PPE infusion was performed in 10 to 14-week-old male mice [83]. In brief, after placing temporary ligatures around the proximal and distal aorta, the infrarenal aorta was infused with elastase from porcine pancreas (E1250, Sigma) for 5 min at 100 mmHg. Body temperature was kept constantly at 37 °C by a heating pad. After removing the infusion catheter, the aortotomy was sutured, and the abdomen was closed. The abdominal segments were harvested at 3- or 28-day post-PPE surgery. For histological and immunofluorescence stainings, the aorta was perfused with saline followed by 1.5% agarose before harvesting. For RNA isolation, the aorta was collected in TRIzol™ Reagent (15596026, ThermoFischer Scientific).

### Transmission electron microscopy

The abdominal aorta was harvested 28 days post-PPE. The tissue was fixed in TEM fixative containing 2% glutaraldehyde and 2% formaldehyde in 0.1 M cacodylate buffer (pH 7.3) for 48 h, then washed with 0.1 M cacodylate buffer (pH 7.3). After washing, postfixation was applied using 2% OsO4 (Science Services) in 0.1 M cacodylate buffer for 2 h at 4 °C followed by washing for four times with 0.1 M cacodylate buffer and dehydration in graded ethanol series (1 × 50%, 1 × 70%, 1 × 90%, 3 × 100%) for 15 min each. Samples were then incubated in a mix of 50% ethanol/propylenoxide, then two times with pure propylenoxide for 15 min each. Samples were infiltrated with a mixture of 50% epon/propylenoxide and 75% epon/propylenoxide for 2 h each at 4 °C and with pure Epon overnight at 4 °C. The next day, epon was exchanged, and samples were incubated for 2 h at RT, placed into TAAB capsules and cured for 72 h at 60 °C. Ultrathin (~70 nm) sections were obtained using Leica EM UC6 Ultramicrotome (Leica, Germany) attached with diamond knife (DiATOME, Switzerland) and double-stained with 1.5% uranyl acetate (15 min at 37 °C) followed by 3% lead citrate for 4 min. Images were acquired using a JEM-2100 Plus Transmission Electron Microscope (JEOL) operating at 80 kV equipped with a OneView 4 K camera (Gatan).

### Ultrasound imaging of the abdominal aorta

To obtain ultrasound images of the abdominal aorta, the mice were anesthetized with continuously delivered 2% isoflurane gas inhalation. Ultrasound gel was applied to the depilated skin of the abdomen, and imaging was performed with a Vevo3100 imaging system (VisualSonics). B-mode, M-mode and EKV recordings were performed using an MX550D linear array transducer (25-55 MHz, Centre Transmit: 40 MHz, Axial Resolution: 40 μm) with a frame rate of 230–400 frames/s. All ultrasound images were analyzed using the Vevo 3100 software, and parameters like aortic diameter, pulse wave velocity (PWV) and aortic wall thickness were calculated.

### Immunofluorescence staining

Abdominal aorta was harvested, cleaned and embedded into Tissue-Tek O.C.T. Compound (Sakura Finetek™ 4583) and stored at –80 °C for cryopreservation. The tissue was cut into 10 µm-thick sections using a Leica CM3050 S Cryostat (Leica, Germany). For Immunofluorescence stainings, the tissue sections were fixed with 4% paraformaldehyde (PFA) for 10 min and permeabilized using 0.1% Triton X-100 for 10 min at room temperature, followed by blocking using 3% bovine serum albumin (BSA) for 1 h at room temperature. Tissue sections were incubated with the primary antibody for anti-alpha smooth muscle Actin antibody (ab5694, Abcam) at 4 °C overnight. Normal rabbit IgG (Thermo Scientific, Waltham, MA, USA, 026102) was used for the negative controls. Next day, the sections were washed three times in PBS followed by incubation with Goat anti-Rabbit IgG (H + L) Cross-Adsorbed Secondary Antibody, Alexa Fluor™ 488 (A-11008, Invitrogen) and Alexa Fluor^®^ 594 anti-mouse CD68 Antibody (137020, BioLegend, USA) or Alexa Fluor^®^ 594 anti-mouse Ly-6G Antibody (127636, BioLegend, USA) for 1 h at room temperature in the dark followed by washing and mounting. Cell nuclei were stained with DAPI. Images were acquired with a BZ-X800 microscope system (Keyence, USA) and analyzed using ImageJ software.

### Histology

Masson Trichrome staining was performed using Trichrome Stain (Masson) Kit (HT15, Sigma) according to the manufacturer’s protocol. Images were acquired with a BZ-X800 microscope system (Keyence, USA) and analyzed using ImageJ software.

For elastin fibers, the tissue sections were imaged for the elastin autofluorescence using the GFP filter on BZ-X800 microscope system (Keyence, USA) and analyzed using ImageJ software. The processed images were graded as per the grading system outlined in Supplementary Fig. S2A, B for MTS and elastin grading, respectively [84].

### Flow cytometry analysis

Mice under 3% Isoflurane anesthesia were surgically opened, perfused with 10 ml PBS (0.5 mM EDTA) via left ventricle. Abdominal aorta was isolated and digested for 1 h, 37 °C, 45 rpm agitation in HBSS with 450U/ml Collagenase I (Sigma), 125U/ml Collagenase XI (Sigma) 60U/ml Hyalurase (Sigma), 60U/ml DNAse1 (ThermoFisher) and filtered through 100 µm cell strainers (Sysmex) to achieve a single cell suspension. Samples were treated with FcR block (TruStain FcX, Biolegend, 1:100) and live-dead staining (Zombie UV, Biolegend) according to manufacturer’s protocol. Extracellular stainings were performed in FACS buffer, 20 min, 4 °C using the antibodies listed in the Table 1. Samples were acquired on an Aurora 5 L Flow cytometer (Cytek Biosciences), unmixing and spillover correction were performed on SpectroFlo (Cytek Biosciences), conventional FCS gating and clustering were performed in Flowjo 10.8.1 with Phenograph and UMAP plugins. Samples of low cellularity were excluded from further analysis.

**Table 1:** Antibodies used for flow cytometry (FACS) analysis.

Antigen	Fluor	Clone	Dilution	Manufacturer
**CD45**	BUV496	30-F11	1:200	BD
Ly6G	BUV563	1-A8	1:200	BD
CD11b	BUV661	M1/70	1:400	BD
CD3e	BUV737	145-2C11	1:150	BD
MeRTK	BV421	2B10C42	1:100	Biolegend
CD11c	Pacific Blue	N418	1:200	Biolegend
Ly6C	BV650	HK1.4	1:100	Biolegend
CD16.2	BV711	9.00E + 09	1:100	Biolegend
CD8a	BV750	53-6.7	1:200	Biolegend
CCR2	BV786	SA203G11	1:100	Biolegend
CD115	AF488	AFS98	1:100	Biolegend
CD4	Spark Blue 550	GK1.5	1:200	Biolegend
TIMD4	PE	RMT4-54	1:100	Biolegend
F4/80	PerCP/Cy5.5	BM8	1:100	Biolegend
Lyve-1	PE/Cy7	ALY-7	1:100	Thermo
CD64	APC	X54-5/7.1	1:100	Biolegend
CD19	Spark NIR 685	6D5	1:400	Biolegend
MHCII	AF700	M5/114.15.2	1:100	Biolegend
CX3CR1	APC/Fire750	SA011F11	1:200	Biolegend

Immune cell populations were identified as CD45^+^, zombieUV^-^. Subpopulations were defined as Neutrophils (CD11b^+^, CD3^-^, Ly6G^+^); B-Cells (CD11b^-^, CD3^-^, CD19^+^, MHCII^+^); conventional T-cells (either CD11b^-^, CD3^+^, CD4^+^ or CD11b^-^, CD3^+^, CD8^+^); M1-macrophages (CD11b^+^, CD3^-^, F4/80^+^, MHCII^+^) or M2-macrophages (CD11b^+^, CD3^-^, F4/80^+^, MHCII^-^). Significant changes in cellular composition between the WT and *Mlkl*^*–/–*^ were assessed by Welch ANOVA and Brown-Forsythe test in Prism9 (GraphPad Software). Abdominal aortic samples were further analyzed by UMAP and stratified according to surface marker expression.

### mRNAseq analysis

Total RNA was extracted from cleaned abdominal aortas 3 days post-PPE from WT, *Ripk1*^*D138N/D138N*^, *Mlkl*^*AA*^ and *Mlkl*^*–/–*^ animals (*n* = 4 each) and abdominal aortae from untreated WT (WT baseline) animals were used as control. RNA was shipped to Novogene (Cambridge, UK), where the mRNA sequencing was performed as per their in-house protocols. Gene expression read counts were exported and analyzed using the integrated Differential Expression and Pathway (iDEP) tool (http://bioinformatics.sdstate.edu/idep96/) to identify the differentially expressed genes (DEGs) and enriched GO terms, results were visualized with GOplot package [85] volcano plots are created using VolcaNoseR web application[86].

### Bone marrow transplantation

In a bone marrow (BM) transplantation study, irradiated recipient WT mice were reconstituted with *Mlkl*^*AA*^-BM (WT^MLKLAA-BM^) and irradiated *Mlkl*^*AA*^ recipient mice were reconstituted with WT-BM (MLKL^AA+WT-BM^), resulting in SMC (*WT*)/ macrophage (*Mlkl*^*AA*^) and SMC (*Mlkl*^*AA*^)/ macrophage (WT) chimera mice. Mice were given neomycin (1.6 mg/ml) through drinking water 8 days before the irradiation. Recipient mice were irradiated with10 Gy whole-body irradiation in a Cesium-137 gamma source (Biobeam GM 8000). Bone marrow cells from the donor animals were isolated from the femurs and resuspended in cold PBS, and 5 × 10^6^ bone marrow cells (150 μL) were injected by tail vein into each of the recipient mice 24 h after irradiation. Four weeks after transplantation, a small amount of blood was withdrawn, and platelet counts were determined by using Element HT5 (scil animal care company GmbH, Germany), and animals were subjected to PPE surgery. Transplantation efficacy was determined by flow cytometry for CD45.1 (donor) and CD45.2 (recipient) 5 days before PPE and reached 95% cell reconstitution in all animals.

## In vitro methods

### Reagents

All the cell culture reagents, such as DMEM, RPMI, puromycin, TrypLE™ select enzyme, FBS, HBSS buffer, etc., were purchased from Gibco/Thermo Scientific (Waltham, MA, USA). Necrosulfonamide was purchased from Tocris (Cat. No. 5025/10). Elastase from porcine pancreas was purchased from Sigma (Cat. No. E1250). Doxycycline Hydrochloride as purchased from Sigma (Cat. No. D3072-1ML).

### Cell culture

Human aortic SMC (HAoSMC) were purchased from ATCC (PCS-100-012, ATCC) and propagated in Vascular Cell Basal Medium (PCS-100-030, ATCC) supplemented with Vascular Smooth Muscle Cell Growth Kit (PCS-100-042, ATCC) as per the manufacturer's protocol in a standard cell culture incubator at 5% CO_2_, 37 °C.

HL-60 cells were maintained in RPMI-1640 (72400047, Gibco) with 10% FBS (F9665, Sigma), 1x GlutaMAX™ Supplement (35050061, Gibco), 1x Penicillin-streptomycin (15140122, Gibco) in a 5% CO_2_ atmosphere at 37 °C. The HL-60 cells were differentiated into a polymorphonuclear neutrophil-like (dPMN) phenotype by culturing in RPMI-1640 medium supplemented with 10% FBS and 1.3% DMSO for 6 days [87]. Presence of neutrophil-like cells was confirmed CD11b IF staining and RT-PCR analysis of *Cd11b* transcript.

### MLKL cloning and generation of stable SMC lines

To induce necroptosis in SMCs, we opted for an inducible MLKL overexpression system. The full-length MLKL and N-domain MLKL (amino acid 1–182) were amplified from cDNA clone (RC213152, Origene, USA) using a two-step PCR reaction with primers mentioned in Table 2. The primers are designed to incorporate the attL1 and attL2 overhangs on either side of the PCR product to facilitate the ligation using LR-Clonase enzyme. The PCR product was then ligated into pB-TAC-ERP2 plasmid (PB-TAC-ERP2 was a gift from Knut Woltjen (Addgene plasmid # 80478)) and positive clones were confirmed by DNA sequence analysis.

**Table 2:** Primer sequences were applied to amplify full-length and N-domain (AA 1-183) of hMLKL gene using PCR reaction.

Primer Name	Primer sequence (5’→ 3’)
Step1: Forward	AAAGCAGGCTCCTGCAGGACCATGATGGAAAATTTGAAGC
Step1: Reverse(F-hMLKL)	AGAAAGCTGGGTCTCGAGCTACTACTTAGAAAAGGTG
Step1: Reverse(ND-hMLKL)	AGAAAGCTGGGTCTCGAGCTATGGTGGTAAATACTGC
Step2: Forward	CCCCTTTTATAATGCCAACTTTGTACAAAAAAGCAGGCTCC
Step2: Reverse	GGGGTCTTATAATGCCAACTTTGTACAAGAAAGCTG

To generate the HAoSMC lines overexpressing hMLKL, the cells were transfected with donor pB-TAC-ERP2 vector containing either full-length hMLKL (F-hMLKL) or N-domain hMLKL (ND-hMLKL) isoform, along with pCAGPBase plasmid (pCAGPBase was a gift from Joseph Loturco (Addgene plasmid # 40972)) encoding for transposase. 48 h post-transfection, the cells were selected using 2 µg/ml of puromycin. Post-transfection the serum in the growth media was replaced with Tet system-approved serum (A4736101, ThermoFischer). Cells were treated with 1 µg/ml Doxycycline to induce the MLKL overexpression, and for all experiments, the cells between passage three and seven were used.

### Cytokine array

A Cytokine Antibody Array (ab133998 (human), ab133995 (mouse) Abcam was used to analyze the supernatant of SMCs as per the manufacturer’s instructions. In brief, 0.1 × 10^6^ cells were seeded in a 6-well plate and cultured till ~70% confluence. Then necroptosis was induced, and supernatants were collected after 24 h for the measurement of released cytokines. Supernatant from untreated SMCs was used as an uninduced control. The dot blots were used to calculate the integrated densities using ImageJ and “Protein Array Analyzer” Tool [88].

### Transwell migration assay

Polymorphonuclear (PMNs) cells were differentiated from HL60 cells by DMSO treatment for 6 days, referred here as dPMN, and their phenotype was confirmed by staining for CD11b and qRT-PCR. These dPMN cells were subjected to transmigration using 6.5-mm-diameter Transwell Inserts with 5-μm pore size Polyester Membrane (Corning, NY, USA). The inserts were placed into the 24-well plate containing control or SMC-conditioned media, making sure no air bubbles were trapped under the membrane. The dPMN were seeded at a final concentration of 1.5 × 10^6^ cells per well in the top chamber and allowed to transmigrate for 1 h at 37 C°. Following this period, inserts with non-migrated cells were removed, and migrated cells in the bottom chamber were stained with Trypan blue and counted with a Neubauer chamber or Hemocytometer.

### Statistical analysis and statements

Sample sizes were chosen based on established practice in the field and on our previous publications using the elastase-induced AAA model, where similar group sizes provided sufficient power to detect biologically and statistically meaningful differences. Animals were operated in groups of *n* > 4 to reduce operational bias. The exact numbers of animals used per group are provided in the figure legends. Mice that were suffering after surgery, according to the 3 R principle and animal welfare regulations, were excluded from analysis prior to data collection or by using the ROUT outlier test. Animals were randomly assigned to experimental groups at the time of surgery using simple randomization. Subsequent sample processing and analysis were performed in a blinded manner by two independent researchers to minimize personal bias. Data are presented as mean ± SEM. Shapiro-Wilk test and Brown-Forsythe test were utilized to test for normal distribution and equality of variances, respectively. Differences between three or four groups were evaluated using one-way or two-way repeated measures analysis of variance (ANOVA) with post-hoc Tukey’s test if data were normally distributed and variances were equal. Kruskal–Wallis test with Dunn’s post-hoc test was performed if data were not normally distributed. For comparison of two data sets consisting of normally distributed data, the unpaired t-test was utilized. A *P*-value < 0.05 was considered statistically significant. All statistical analyses were performed using GraphPad Prism 8.4.0 (GraphPad Software, San Diego, CA, USA).

## Supplementary information

Supplementary Materials

Original Data

Supplementary data_Bulk mRNA seq

Supplementary_Video_1

Supplementary_Video_2

## Data Availability

The bulk RNA-seq data generated during this study are included in the published article and its supplementary information files.

## References

[1] Li X, Zhao G, Zhang J, Duan Z, Xin S. Prevalence and trends of the abdominal aortic aneurysms epidemic in general population—a meta-analysis. PLoS ONE. 2013;8:e81260. 24312543 10.1371/journal.pone.0081260 PMC3846841

[2] Parkinson F, Ferguson S, Lewis P, Williams IM, Twine CP. Rupture rates of untreated large abdominal aortic aneurysms in patients unfit for elective repair. J Vasc Surg. 2015;61:1606–12. 25661721 10.1016/j.jvs.2014.10.023

[3] Henderson EL, Geng Y-J, Sukhova GK, Whittemore AD, Knox J, Libby P. Death of smooth muscle cells and expression of mediators of apoptosis by T lymphocytes in human abdominal aortic aneurysms. Circulation. 1999;99:96–104. 9884385 10.1161/01.cir.99.1.96

[4] Singh R, Letai A, Sarosiek K. Regulation of apoptosis in health and disease: the balancing act of BCL-2 family proteins. Nat Rev Mol Cell Biol. 2019;20:175–93. 30655609 10.1038/s41580-018-0089-8 PMC7325303

[5] Fink SL, Cookson BT. Apoptosis, pyroptosis, and necrosis: mechanistic description of dead and dying eukaryotic cells. Infect Immun. 2005;73:1907–16. 15784530 10.1128/IAI.73.4.1907-1916.2005 PMC1087413

[6] Pasparakis M, Vandenabeele P. Necroptosis and its role in inflammation. Nature. 2015;517:311–20. 25592536 10.1038/nature14191

[7] Dai W, Cheng J, Leng X, Hu X, Ao Y. The potential role of necroptosis in clinical diseases (Review). Int J Mol Med. 2021;47:89. 33786617 10.3892/ijmm.2021.4922 PMC8012024

[8] Garcia LR, Tenev T, Newman R, Haich RO, Liccardi G, John SW, et al. Ubiquitylation of MLKL at lysine 219 positively regulates necroptosis-induced tissue injury and pathogen clearance. Nat Commun. 2021;12:3364. 34099649 10.1038/s41467-021-23474-5 PMC8184782

[9] Cai Z, Jitkaew S, Zhao J, Chiang H-C, Choksi S, Liu J, et al. Plasma membrane translocation of trimerized MLKL protein is required for TNF-induced necroptosis. Nat Cell Biol. 2014;16:55–65. 24316671 10.1038/ncb2883 PMC8369836

[10] Choi ME, Price DR, Ryter SW, Choi AMK. Necroptosis: a crucial pathogenic mediator of human disease. JCI Insight 2019;4. 10.1172/jci.insight.128834. 10.1172/jci.insight.128834 PMC6693822 31391333

[11] Kaczmarek A, Vandenabeele P, Krysko DV. Necroptosis: the release of damage-associated molecular patterns and its physiological relevance. Immunity. 2013;38:209–23. 23438821 10.1016/j.immuni.2013.02.003

[12] Shi J, Guo J, Li Z, Xu B, Miyata M. Importance of NLRP3 inflammasome in abdominal aortic aneurysms. J Atheroscler Thromb. 2021;28:454–66. 33678767 10.5551/jat.RV17048 PMC8193780

[13] Roh JS, Sohn DH. Damage-associated molecular patterns in inflammatory diseases. Immune Netw. 2018;18:e27. 30181915 10.4110/in.2018.18.e27 PMC6117512

[14] Eliason JL, Hannawa KK, Ailawadi G, Sinha I, Ford JW, Deogracias MP, et al. Neutrophil depletion inhibits experimental abdominal aortic aneurysm formation. Circulation. 2005;112:232–40. 16009808 10.1161/CIRCULATIONAHA.104.517391

[15] Isoda K, Akita K, Kitamura K, Sato-Okabayashi Y, Kadoguchi T, Isobe S, et al. Inhibition of interleukin-1 suppresses angiotensin II-induced aortic inflammation and aneurysm formation. Int J Cardiol. 2018;270:221–7. 29884291 10.1016/j.ijcard.2018.05.072

[16] Ridker PM, Everett BM, Thuren T, MacFadyen JG, Chang WH, Ballantyne C, et al. Antiinflammatory therapy with canakinumab for atherosclerotic disease. N Engl J Med. 2017;377:1119–31. 28845751 10.1056/NEJMoa1707914

[17] Tardif J-C, Kouz S, Waters DD, Bertrand OF, Diaz R, Maggioni AP, et al. Efficacy and safety of low-dose colchicine after myocardial infarction. N Engl J Med. 2019;381:2497–505. 31733140 10.1056/NEJMoa1912388

[18] Golledge J, Moxon JV, Singh TP, Bown MJ, Mani K, Wanhainen A. Lack of an effective drug therapy for abdominal aortic aneurysm. J Intern Med. 2020;288:6–22. 31278799 10.1111/joim.12958

[19] Wang Q, Liu Z, Ren J, Morgan S, Assa C, Liu B. Receptor-interacting protein kinase 3 contributes to abdominal aortic aneurysms via smooth muscle cell necrosis and inflammation. Circ Res. 2015;116:600–11. 25563840 10.1161/CIRCRESAHA.116.304899 PMC4329096

[20] Wang Q, Zhou T, Liu Z, Ren J, Phan N, Gupta K, et al. Inhibition of Receptor-interacting protein kinase 1 with necrostatin–1s ameliorates disease progression in elastase-induced mouse abdominal aortic aneurysm model. Sci Rep. 2017;7:42159. 28186202 10.1038/srep42159 PMC5301478

[21] Biros E, Gäbel G, Moran CS, Schreurs C, Lindeman JHN, Walker PJ, et al. Differential gene expression in human abdominal aortic aneurysm and aortic occlusive disease. Oncotarget. 2015;6:12984–96. 10.18632/oncotarget.3848 PMC4536993 25944698

[22] Jing J, Yang IV, Hui L, Patel JA, Evans CM, Prikeris R, et al. Role of macrophage receptor with collagenous structure in innate immune tolerance. J Immunol. 2013;190:6360–7. 23667110 10.4049/jimmunol.1202942 PMC3679202

[23] Ghosh S, Gregory D, Smith A, Kobzik L. MARCO regulates early inflammatory responses against influenza: a useful macrophage function with adverse outcome. Am J Respir Cell Mol Biol. 2011;45:1036–44. 21562316 10.1165/rcmb.2010-0349OC PMC3262690

[24] Son B-K, Sawaki D, Tomida S, Fujita D, Aizawa K, Aoki H, et al. Granulocyte macrophage colony-stimulating factor is required for aortic dissection/intramural haematoma. Nat Commun. 2015;6:6994. 25923510 10.1038/ncomms7994

[25] Maegdefessel L, Spin JM, Raaz U, Eken SM, Toh R, Azuma J, et al. miR-24 limits aortic vascular inflammation and murine abdominal aneurysm development. Nat Commun. 2014;5:5214. 25358394 10.1038/ncomms6214 PMC4217126

[26] Liu W, Liu Y, Wang R, Li C, Deng C, Rodgers GP. Olfactomedin 4 is essential for superoxide production and sensitizes oxidative stress-induced apoptosis in neutrophils. Blood. 2009;114:1356.

[27] Ren X, Geng M, Xu K, Lu C, Cheng Y, Kong L, et al. Quantitative proteomic analysis of synovial tissue reveals that upregulated OLFM4 aggravates inflammation in rheumatoid arthritis. J Proteome Res. 2021;20:4746–57. 34496567 10.1021/acs.jproteome.1c00399

[28] Houard X, Touat Z, Ollivier V, Louedec L, Philippe M, Sebbag U, et al. Mediators of neutrophil recruitment in human abdominal aortic aneurysms. Cardiovasc Res. 2009;82:532–41. 19201759 10.1093/cvr/cvp048 PMC2682614

[29] Quarato G, Guy CS, Grace CR, Llambi F, Nourse A, Rodriguez DA, et al. Sequential engagement of distinct MLKL phosphatidylinositol-binding sites executes necroptosis. Mol Cell. 2016;61:589–601. 26853145 10.1016/j.molcel.2016.01.011 PMC4769881

[30] Meher AK, Spinosa M, Davis JP, Pope N, Laubach VE, Su G, et al. Novel role of IL (Interleukin)-1β in neutrophil extracellular trap formation and abdominal aortic aneurysms. Arterioscler Thromb Vasc Biol. 2018;38:843–53. 29472233 10.1161/ATVBAHA.117.309897 PMC5864548

[31] López-Candales A, Holmes DR, Liao S, Scott MJ, Wickline SA, Thompson RW. Decreased vascular smooth muscle cell density in medial degeneration of human abdominal aortic aneurysms. Am J Pathol. 1997;150:993–1007. 9060837 PMC1857880

[32] Allaire E, Muscatelli-Groux B, Mandet C, Guinault A-M, Bruneval P, Desgranges P, et al. Paracrine effect of vascular smooth muscle cells in the prevention of aortic aneurysm formation. J Vasc Surg. 2002;36:1018–26. 12422114 10.1067/mva.2002.127347

[33] Chen Y, He Y, Wei X, Jiang D-S. Targeting regulated cell death in aortic aneurysm and dissection therapy. Pharmacol Res. 2022;176:106048. 34968685 10.1016/j.phrs.2021.106048

[34] Newton K. Multitasking kinase RIPK1 regulates cell death and inflammation. Cold Spring Harb Perspect Biol 2020;12. 10.1101/cshperspect.a036368. 10.1101/cshperspect.a036368 PMC7050590 31427374

[35] Rodriguez DA, Weinlich R, Brown S, Guy C, Fitzgerald P, Dillon CP, et al. Characterization of RIPK3-mediated phosphorylation of the activation loop of MLKL during necroptosis. Cell Death Differ. 2016;23:76–88. 26024392 10.1038/cdd.2015.70 PMC4815980

[36] Laurien L, Nagata M, Schünke H, Delanghe T, Wiederstein JL, Kumari S, et al. Autophosphorylation at serine 166 regulates RIP kinase 1-mediated cell death and inflammation. Nat Commun. 2020;11:1747. 32269263 10.1038/s41467-020-15466-8 PMC7142081

[37] Ang LS, Boivin WA, Williams SJ, Zhao H, Abraham T, Carmine-Simmen K, et al. Serpina3n attenuates granzyme B-mediated decorin cleavage and rupture in a murine model of aortic aneurysm. Cell Death Dis. 2011;2:e209–e209. *2011 2:9*. 21900960 10.1038/cddis.2011.88 PMC3186906

[38] Zheng Y, Gardner SE, Clarke MCH. Cell death, damage-associated molecular patterns, and sterile inflammation in cardiovascular disease. Arterioscler Thromb Vasc Biol. 2011;31:2781–6. 22096097 10.1161/ATVBAHA.111.224907

[39] Gong T, Liu L, Jiang W, Zhou R. DAMP-sensing receptors in sterile inflammation and inflammatory diseases. Nat Rev Immunol. 2020;20:95–112. 31558839 10.1038/s41577-019-0215-7

[40] Pagano MB, Bartoli MA, Ennis TL, Mao D, Simmons PM, Thompson RW, et al. Critical role of dipeptidyl peptidase I in neutrophil recruitment during the development of experimental abdominal aortic aneurysms. Proc Natl Acad Sci USA. 2007;104:2855–60. 17301245 10.1073/pnas.0606091104 PMC1797622

[41] Yan H, Zhou HF, Akk A, Hu Y, Springer LE, Ennis TL, et al. Neutrophil proteases promote experimental abdominal aortic aneurysm via extracellular trap release and plasmacytoid dendritic cell activation. Arterioscler Thromb Vasc Biol. 2016;36:1660–9. 27283739 10.1161/ATVBAHA.116.307786 PMC4965335

[42] Shimizu K, Mitchell RN, Libby P. Inflammation and cellular immune responses in abdominal aortic aneurysms. Arterioscler Thromb Vasc Biol. 2006;26:987–94. 16497993 10.1161/01.ATV.0000214999.12921.4f

[43] Haider P, Kral-Pointner JB, Mayer J, Richter M, Kaun C, Brostjan C, et al. Neutrophil extracellular trap degradation by differently polarized macrophage subsets. Arterioscler Thromb Vasc Biol. 2020;40:2265–78. 32673525 10.1161/ATVBAHA.120.314883 PMC7447175

[44] Ludwig A, Petersen F, Zahn S, Götze O, Schröder J-M, Flad H-D, et al. The CXC-chemokine neutrophil-activating peptide-2 induces two distinct optima of neutrophil chemotaxis by differential interaction with interleukin-8 receptors CXCR-1 and CXCR-2. Blood. 1997;90:4588–97. 9373270

[45] Owens GK, Kumar MS, Wamhoff BR. Molecular regulation of vascular smooth muscle cell differentiation in development and disease. Physiol Rev. 2004;84:767–801. 15269336 10.1152/physrev.00041.2003

[46] Daugherty A, Manning MW, Cassis LA. Angiotensin II promotes atherosclerotic lesions and aneurysms in apolipoprotein E-deficient mice. J Clin Investig. 2000;105:1605–12. 10841519 10.1172/JCI7818 PMC300846

[47] Lu H, Rateri DL, Cassis LA, Daugherty A. The role of the renin-angiotensin system in aortic aneurysmal diseases. Curr Hypertens Rep. 2008;10:99–106. 18474175 10.1007/s11906-008-0020-3 PMC2846534

[48] Michel JB, Martin-Ventura JL, Egido J, Sakalihasan N, Treska V, Lindholt J, et al. Novel aspects of the pathogenesis of aneurysms of the abdominal aorta in humans. Cardiovasc Res. 2011;90:18–27. 21037321 10.1093/cvr/cvq337 PMC3058728

[49] Satoh K, Nigro P, Matoba T, O’Dell MR, Cui Z, Shi X, et al. Cyclophilin A enhances vascular oxidative stress and the development of angiotensin II–induced aortic aneurysms. Nat Med. 2009;15:649–56. 19430489 10.1038/nm.1958 PMC2704983

[50] Zhu J, Meganathan I, MacAruthur R, Kassiri Z. Inflammation in Abdominal Aortic Aneurysm: cause or co-morbidity? Can J Cardiol. 2024;40:2378–91. 39181326 10.1016/j.cjca.2024.08.274

[51] Vanmaele A, Bouwens E, Hoeks SE, Kindt A, Lamont L, Fioole B, et al. Targeted plasma multi-omics propose glutathione, glycine and serine as biomarkers for abdominal aortic aneurysm growth on serial CT scanning. Atherosclerosis. 2024;398:118620. 39378678 10.1016/j.atherosclerosis.2024.118620

[52] Hinterseher I, Erdman R, Donoso LA, Vrabec TR, Schworer CM, Lillvis JH, et al. Role of complement cascade in abdominal aortic aneurysms. Arterioscler Thromb Vasc Biol. 2011;31:1653–60. 21493888 10.1161/ATVBAHA.111.227652 PMC3712630

[53] Rhee EJ, Nallamshetty S, Plutzky J. Retinoid metabolism and its effects on the vasculature. Biochimica Biophysica Acta. 2012;1821:230–40. 10.1016/j.bbalip.2011.07.001 21810483

[54] Hou Y, Guo W, Fan T, Li B, Ge W, Gao R, et al. Advanced research of abdominal aortic aneurysms on metabolism. Front Cardiovasc Med 2021;8. 10.3389/FCVM.2021.630269. 10.3389/fcvm.2021.630269 PMC7892590 33614752

[55] Skagius E, Siegbahn A, Bergqvist D, Henriksson AE. Fibrinolysis in patients with an abdominal aortic aneurysm with special emphasis on rupture and shock. J Thromb Haemost. 2008;6:147–50. 17922806 10.1111/j.1538-7836.2007.02791.x

[56] Yamazumi K, Ojiro M, Okumura H, Aikou T. An activated state of blood coagulation and fibrinolysis in patients with abdominal aortic aneurysm. Am J Surg. 1998;175:297–301. 9568655 10.1016/s0002-9610(98)00014-2

[57] Murthy S, Larson-Casey JL, Ryan AJ, He C, Kobzik L, Carter AB. Alternative activation of macrophages and pulmonary fibrosis are modulated by scavenger receptor, macrophage receptor with collagenous structure. FASEB J. 2015;29:3527–36. 25953850 10.1096/fj.15-271304 PMC4511206

[58] Zhou G, Zhang L, Shao S. The application of MARCO for immune regulation and treatment. Mol Biol Rep. 2024;51:1–14. 10.1007/s11033-023-09201-x 38300385

[59] Martin KR, Wong HL, Witko-Sarsat V, Wicks IP. G-CSF—A double-edge sword in neutrophil-mediated immunity. Semin Immunol. 2021;54:101516. 34728120 10.1016/j.smim.2021.101516

[60] Middleton RK, Lloyd GM, Bown MJ, Cooper NJ, London NJ, Sayers RD. The pro-inflammatory and chemotactic cytokine microenvironment of the abdominal aortic aneurysm wall: a protein array study. J Vasc Surg. 2007;45:574–80. 17321344 10.1016/j.jvs.2006.11.020

[61] Mulorz J, Spin JM, Mulorz P, Wagenhäuser MU, Deng A, Mattern K, et al. E-cigarette exposure augments murine abdominal aortic aneurysm development: role of Chil1. Cardiovasc Res. 2023;119:867–78. 36413508 10.1093/cvr/cvac173 PMC10409905

[62] Lambert J, Jørgensen HF. Vascular smooth muscle cell phenotypic switching and plaque stability: a role for CHI3L1. Cardiovasc Res. 2021;117:2691–3. 33757119 10.1093/cvr/cvab099 PMC8683704

[63] Chen H, Liu S, Fang G. Knockdown of OLFM4 protects cardiomyocytes from sepsis by inhibiting apoptosis and inflammatory responses. Allergol Immunopathol (Madr). 2024;52:15–20. 39278846 10.15586/aei.v52i5.1145

[64] Gong F, Li R, Zheng X, Chen W, Zheng Y, Yang Z, et al. OLFM4 regulates lung epithelial cell function in sepsis-associated ARDS/ALI via LDHA-mediated NF-κB signaling. J Inflamm Res. 2021;14:7035–51. 34955649 10.2147/JIR.S335915 PMC8694847

[65] Wanhainen A, Verzini F, Van Herzeele I, Allaire E, Bown M, Cohnert T, et al. Editor’s Choice–European Society for Vascular Surgery (ESVS) 2019 Clinical Practice Guidelines on the Management of Abdominal Aorto-iliac Artery Aneurysms. Eur J Vasc Endovasc Surg. 2019;57:8–93. 30528142 10.1016/j.ejvs.2018.09.020

[66] Dubost C, Allary M, Oeconomos N. Resection of an aneurysm of the abdominal aorta: reestablishment of the continuity by a preserved human arterial graft, with result after five months. AMA Arch Surg. 1952;64:405–8. 14894065

[67] Parodi JC, Palmaz JC, Barone HD. Transfemoral intraluminal graft implantation for abdominal aortic aneurysms. Ann Vasc Surg. 1991;5:491–9. 1837729 10.1007/BF02015271

[68] Kayssi A, DeBord Smith A, Roche-Nagle G, Nguyen LL. Health-related quality-of-life outcomes after open versus endovascular abdominal aortic aneurysm repair. J Vasc Surg. 2015;62:491–8. 26211382 10.1016/j.jvs.2015.05.032

[69] Thompson RW, Liao S, Curci JA. Vascular smooth muscle cell apoptosis in abdominal aortic aneurysms. Coron Artery Dis 1997;8:623–31. 10.1097/00019501-199710000-00005 9457444

[70] Zhang J, Schmidt J, Ryschich E, Schumacher H, Allenberg JR. Increased apoptosis and decreased density of medial smooth muscle cells in human abdominal aortic aneurysms. Chin Med J. 2003;116:1549–52. 14570621

[71] Yamanouchi D, Morgan S, Kato K, Lengfeld J, Zhang F, Liu B. Effects of caspase inhibitor on angiotensin II-induced abdominal aortic aneurysm in apolipoprotein E-deficient mice. Arterioscler Thromb Vasc Biol. 2010;30:702–7. 20075419 10.1161/ATVBAHA.109.200527

[72] Jia L-X, Zhang W-M, Li T-T, Liu Y, Piao C-M, Ma Y-C, et al. ER stress-dependent microparticles derived from smooth muscle cells promote endothelial dysfunction during thoracic aortic aneurysm and dissection. Clin Sci. 2017;131:1287–99. 10.1042/CS20170252 PMC5461939 28468950

[73] Boyle JJ, Weissberg PL, Bennett MR. Human macrophage-induced vascular smooth muscle cell apoptosis requires no enhancement of Fas/Fas-L interactions. Arterioscler Thromb Vasc Biol. 2002;22:1624–30. 12377740 10.1161/01.atv.0000033517.48444.1a

[74] Liu Z, Fitzgerald M, Meisinger T, Batra R, Suh M, Greene H, et al. CD95-ligand contributes to abdominal aortic aneurysm progression by modulating inflammation. Cardiovasc Res. 2019;115:807–18. 30428004 10.1093/cvr/cvy264 PMC6432056

[75] Degterev A, Huang Z, Boyce M, Li Y, Jagtap P, Mizushima N, et al. Chemical inhibitor of nonapoptotic cell death with therapeutic potential for ischemic brain injury. Nat Chem Biol. 2005;1:112–9. 16408008 10.1038/nchembio711

[76] Zhou T, Wang Q, Phan N, Ren J, Yang H, Feldman CC, et al. Identification of a novel class of RIP1/RIP3 dual inhibitors that impede cell death and inflammation in mouse abdominal aortic aneurysm models. Cell Death Dis. 2019;10:226. 30842407 10.1038/s41419-019-1468-6 PMC6403222

[77] Cao L, Mu W. Necrostatin-1 and necroptosis inhibition: pathophysiology and therapeutic implications. Pharmacol Res. 2021;163:105297. 33181319 10.1016/j.phrs.2020.105297 PMC7962892

[78] Martens S, Hofmans S, Declercq W, Augustyns K, Vandenabeele P. Inhibitors targeting RIPK1/RIPK3: old and new drugs. Trends Pharmacol Sci. 2020;41:209–24. 32035657 10.1016/j.tips.2020.01.002

[79] Liu F, Wei T, Liu L, Hou F, Xu C, Guo H, et al. Role of necroptosis and immune infiltration in human Stanford type a aortic dissection: novel insights from bioinformatics analyses. Oxid Med Cell Longev 2022;2022. 10.1155/2022/6184802. 10.1155/2022/6184802 PMC9036163 35480868

[80] Zhan C, Huang M, Yang X, Hou J. MLKL: functions beyond serving as the executioner of necroptosis. Theranostics. 2021;11:4759–69. 33754026 10.7150/thno.54072 PMC7978304

[81] Polykratis A, Hermance N, Zelic M, Roderick J, Kim C, Van T-M, et al. Cutting edge: RIPK1 kinase inactive mice are viable and protected from TNF-induced necroptosis in vivo. J Immunol. 2014;193:1539–43. 25015821 10.4049/jimmunol.1400590 PMC4119562

[82] Koerner L, Wachsmuth L, Kumari S, Schwarzer R, Wagner T, Jiao H, et al. ZBP1 causes inflammation by inducing RIPK3-mediated necroptosis and RIPK1 kinase activity-independent apoptosis. Cell Death Differ. 2024;31:938–53. 38849574 10.1038/s41418-024-01321-6 PMC11239871

[83] Azuma J, Asagami T, Dalman R, Tsao PS. Creation of murine experimental abdominal aortic aneurysms with elastase. J Vis Exp 2009. 10.3791/1280. 10.3791/1280 PMC3148686 19629030

[84] Sun J, Sukhova GK, Yang M, Wolters PJ, MacFarlane LA, Libby P, et al. Mast cells modulate the pathogenesis of elastase-induced abdominal aortic aneurysms in mice. J Clin Investig. 2007;117:3359–68. 17932568 10.1172/JCI31311 PMC2000808

[85] Walter W, Sánchez-Cabo F, Ricote M. GOplot: an R package for visually combining expression data with functional analysis. Bioinformatics. 2015;31:2912–4. 25964631 10.1093/bioinformatics/btv300

[86] Goedhart J, Luijsterburg MS. VolcaNoseR is a web app for creating, exploring, labeling and sharing volcano plots. Sci Rep. 2020;10:20560. 33239692 10.1038/s41598-020-76603-3 PMC7689420

[87] Jacob C, Leport M, Szilagyi C, Allen JM, Bertrand C, Lagente V. DMSO-treated HL60 cells: a model of neutrophil-like cells mainly expressing PDE4B subtype. Int Immunopharmacol. 2002;2:1647–56. 12469939 10.1016/s1567-5769(02)00141-8

[88] Watanabe M, Guo W, Zou S, Sugiyo S, Dubner R, Ren K. Antibody array analysis of peripheral and blood cytokine levels in rats after masseter inflammation. Neurosci Lett. 2005;382:128–33. 15911135 10.1016/j.neulet.2005.03.002

