# Supplementary material for: Loss of MLKL impairs abdominal aortic aneurysm development by attenuating smooth muscle cell necroptosis
Source: Cell Death Dis. 2026 Feb 11;17(1):217. doi: 10.1038/s41419-026-08427-4 (PMC12920694; doi:10.1038/s41419-026-08427-4)
Supplement: Supplementary file 1 — Supplementary Materials [file 41419_2026_8427_MOESM1_ESM.pdf]

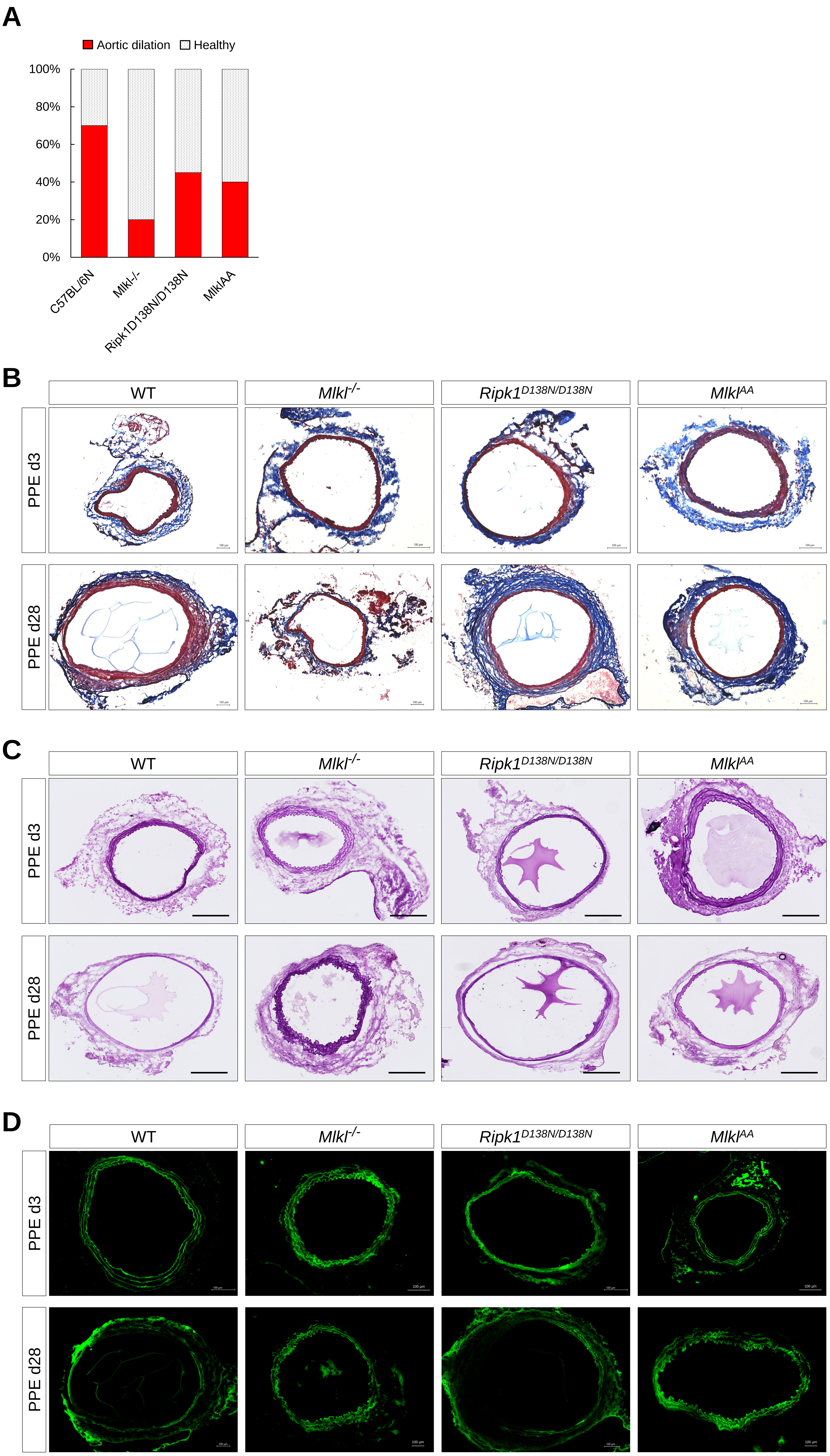

Supplemental Figure S1: (**A**) Occurrence of aortic dilation up to 28 days after PPE in the indicated genotypes. (**B, C and D**) Representative macroscopic images of complete abdominal aortic rings from d3 and d28 PPE animals stained with Masson's Trichrome Staining (MTS) (**B**) and van Gieson's staining (dark blue/purple; top panel) (**C**) and elastin autofluorescence (green; bottom panel) (**D**). Scale bar = 100µm. Related to Figure 1.

A

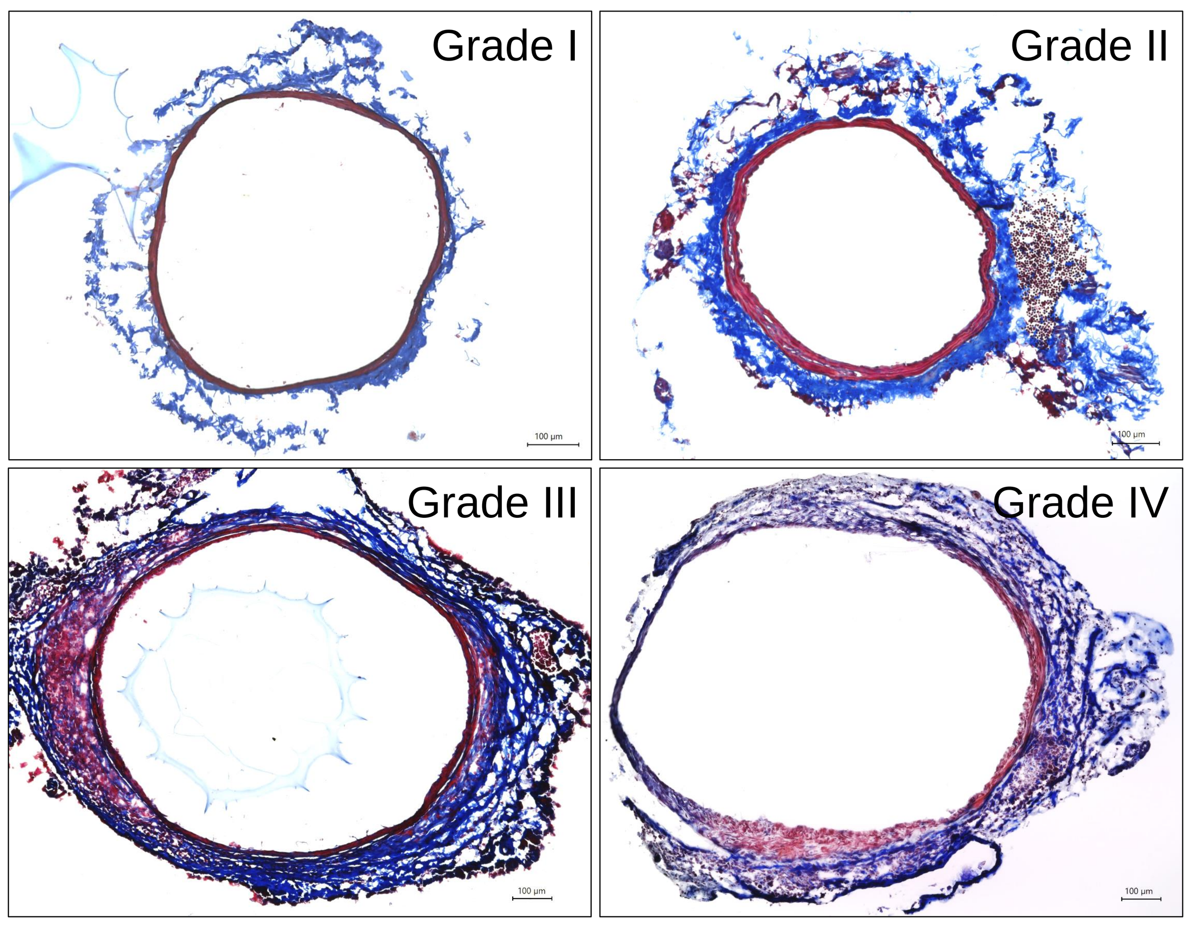

B

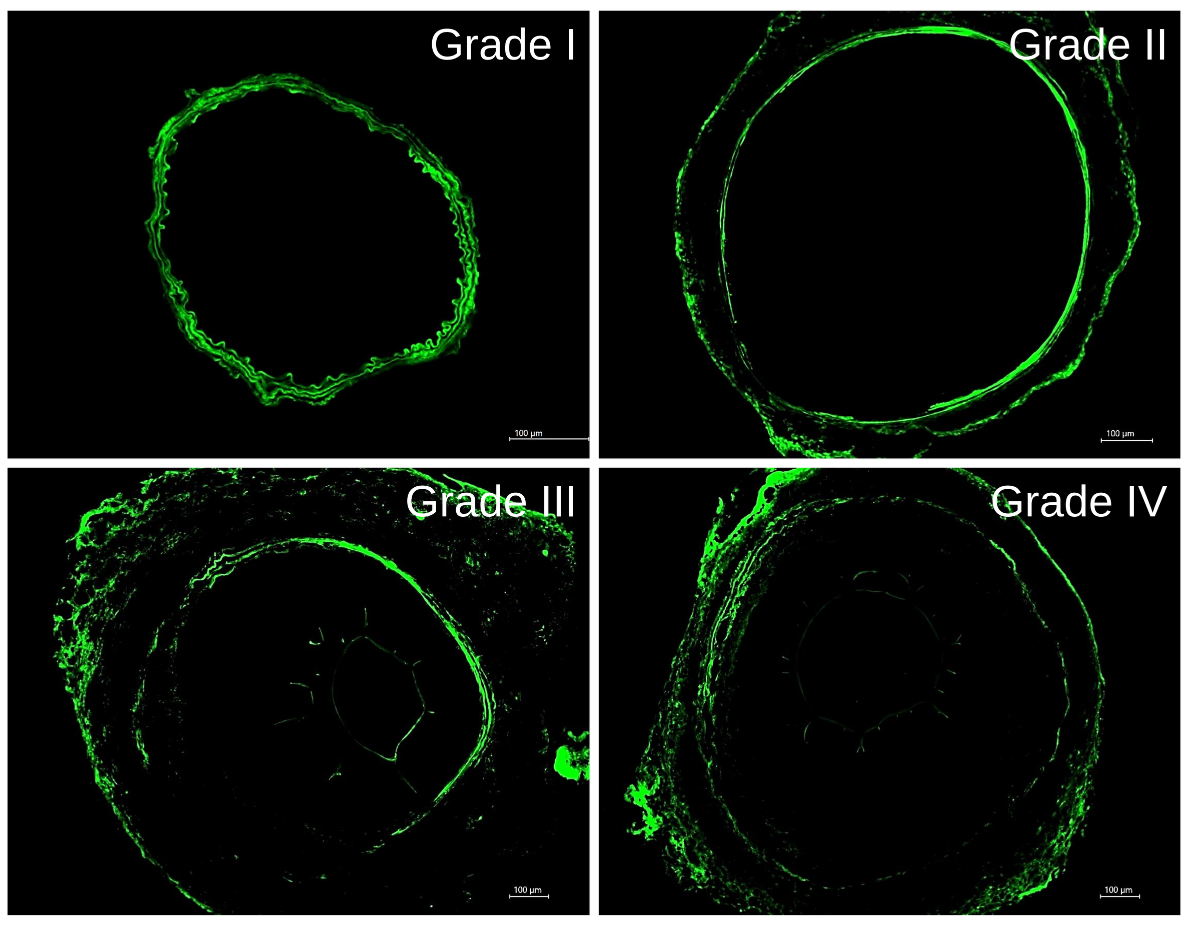

MTS grading	
Grade I	Intact media, wavy collagen fibers bundles (Unoperated (Control) aorta)
Grade II	Small increase in the media content, loss of waviness in collagen fibers bundles
Grade III	Significant increase in the media content and loss of media boundary, loss of waviness in collagen fibers bundles
Grade IV	Significant increase in the media content and loss/ rupture of media boundary, loss of waviness in collagen fibers bundles

Elastin degradation grading	
Grade I	Intact/ wavy elastin fibers (Unoperated (Control) aorta)
Grade II	Visible elastin breaks, loss of waviness in elastin morphology
Grade III	Visible elastin breaks, loss of ~50% of elastin signal
Grade IV	Visible elastin breaks, loss of >50% of elastin signal

C

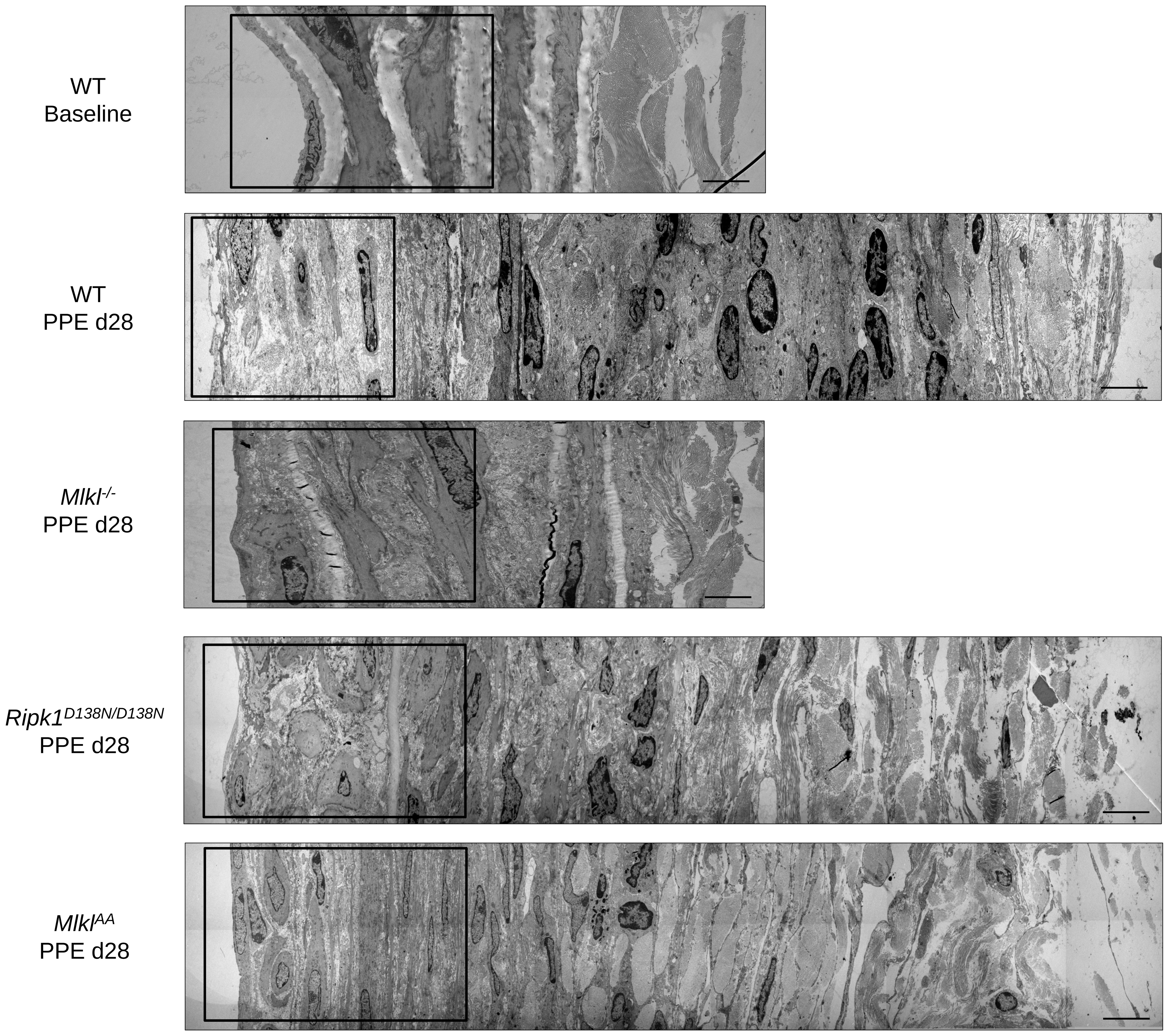

Supplemental Figure S2: Definition and representative images of; (A) Masson's Trichrome Staining (MTS) and (B) elastin degradation grade. (C) Representative transmission electron microscopy (TEM) map images of abdominal aortic sections at baseline and 28 days post-PPE. Scale bar = 1000nm. Related to Figure 1.

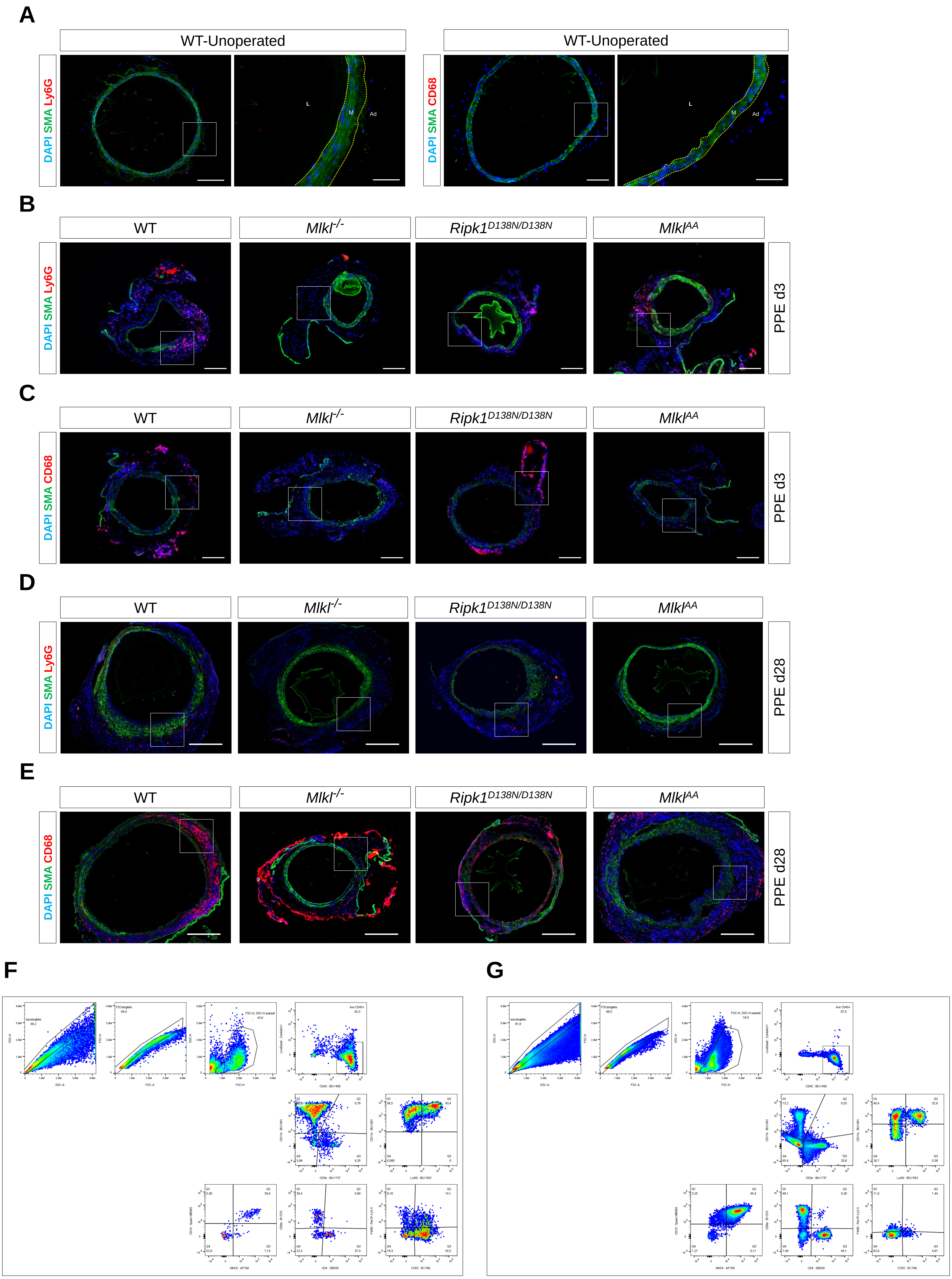

Supplemental Figure S3: Representative confocal images of complete abdominal aortic rings stained with aSMA (green) and Ly6G (red) or CD68 (red) of (**A**) WT baseline aortae, (**B** and **C**) d3 PPE of the indicated genotypes; (**D** and **E**) d28 PPE aortae of the indicated genotypes (scale bars = 100µm and 20µm; Lumen (L), media (M), adventitia (Ad)) (White square indicate image sections shown in the Figure 2 A, C, E and G respectively). (**F** and **G**) Pregating strategy to determine aortic and blood PMN respectively. Related to Figure 2I-K.

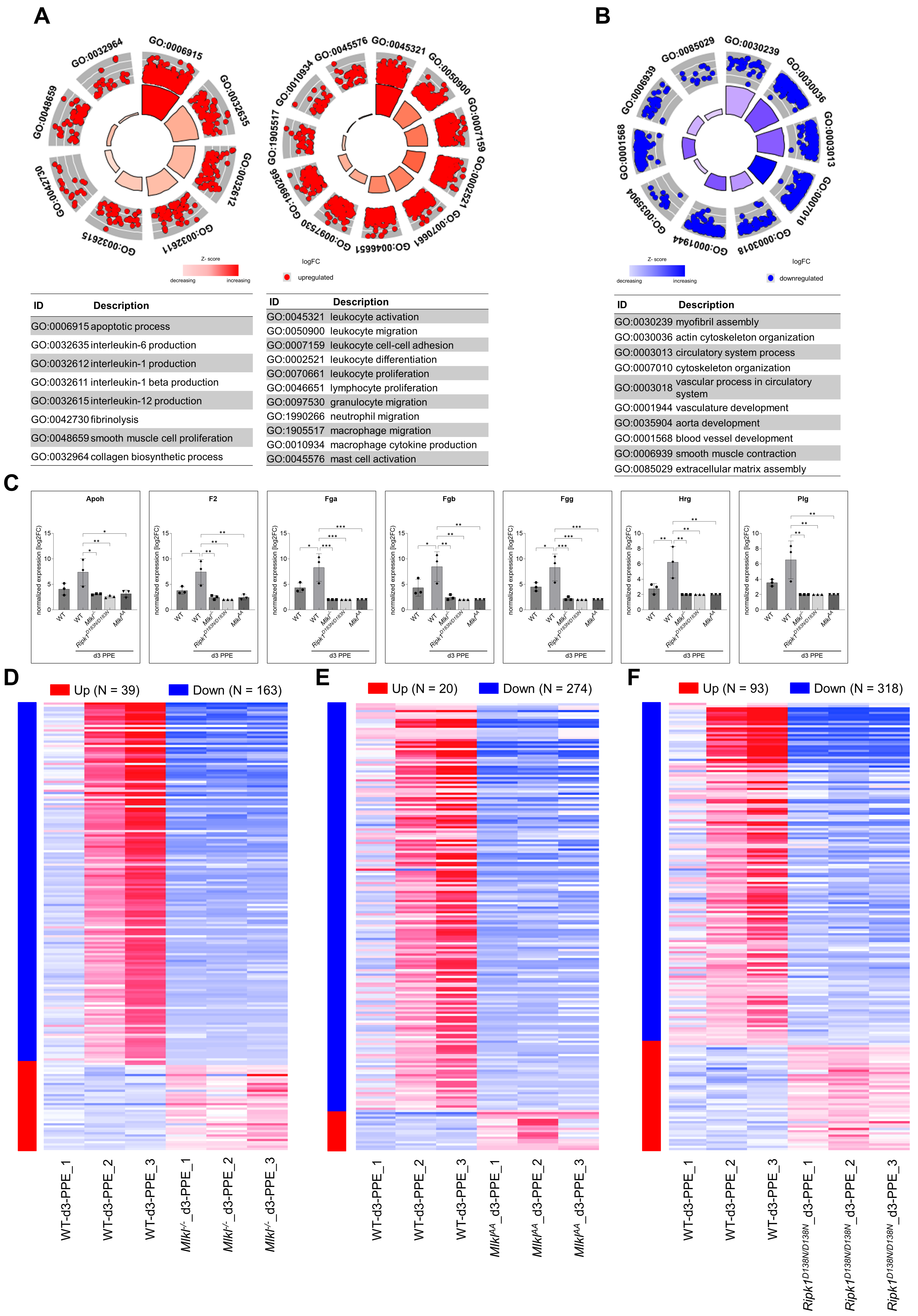

Supplemental Figure S4: (**A** and **B**) GOcircle plots from WT-d3-PPE animals displaying upregulated and downregulated genes with statistically significant GO terms (bottom table) respectively. (**C**) Genes associated with fibrinolysis pathway with their log fold change values from WT-baseline and d3-PPE animals. Data are expressed as mean ± SD (n=3). Statistical significance was determined by Ordinary one-way ANOVA with Tukey's multiple comparisons test. (**D, E** and **F**) Heat map of DEGs from *Mlkl*$^{-/-}$-d3-PPE, *Mlkl*^{AA}-d3-PPE and *Ripk1*$^{D138N/D138N}$-d3-PPE animals respectively. Related to Figure 4A-C.

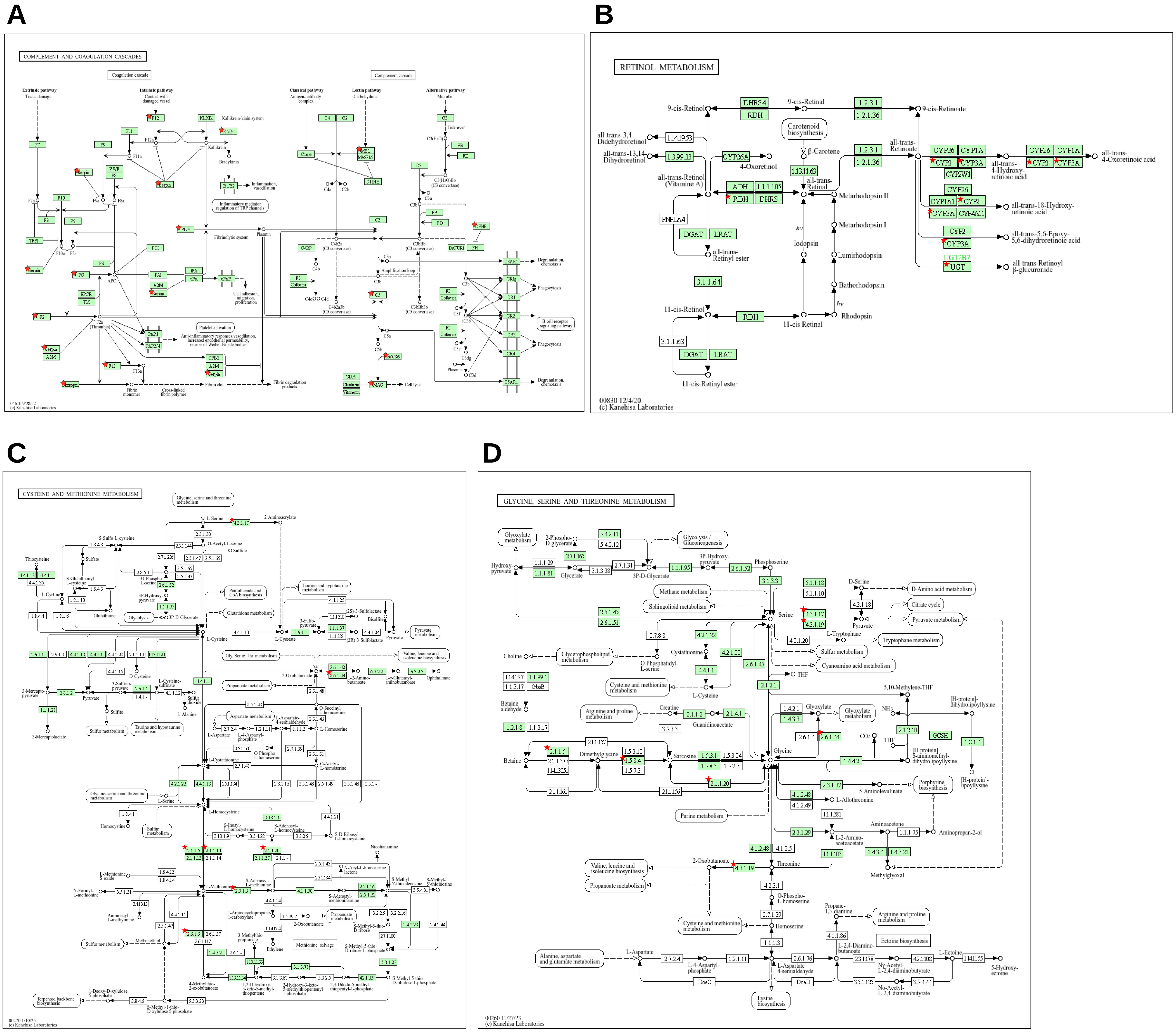

Supplemental Figure S5: Dynamic Visualization of KEGG Pathway maps of the downregulated DEGs implicated in ‘Complement and Coagulation cascades’ (**A**), ‘Retinol metabolism’ (**B**), ‘Cysteine and Methionine metabolism’ (**C**), and ‘Glycine, Serine and Threonine metabolism’ (**D**); the red star marks identified downregulated DEGs. Related to Figure 4D.

A

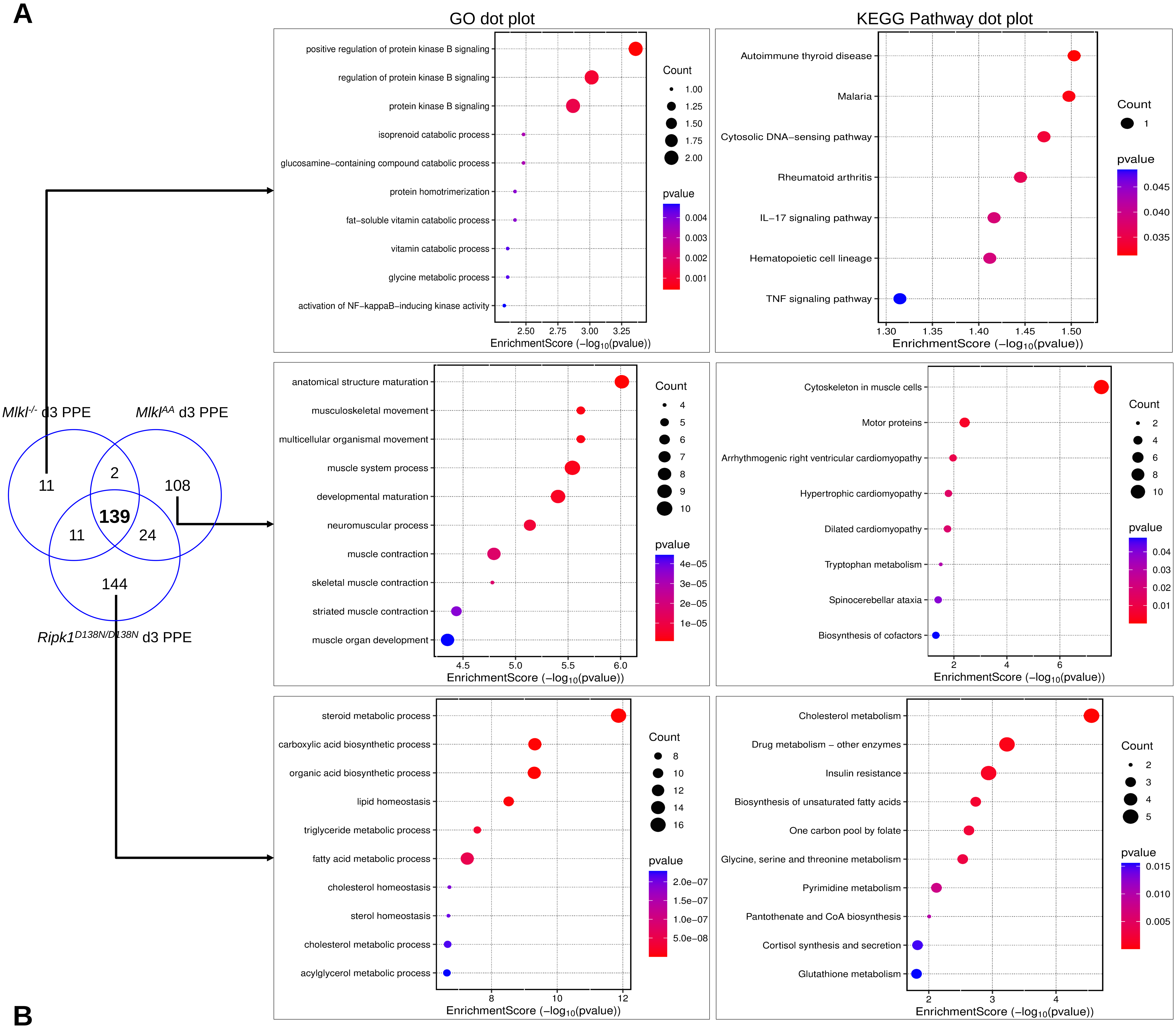

B

Groups	No. Of genes	Gene symbol
Mlkl$^{A/A}$ d3 PPE : Ripk1$^{D138N/D138N}$ d3 PPE	24	Pigr<br>Cyp2a12<br>Cpn2<br>Saa4<br>Serpina11<br>Agxt<br>Aqp8<br>Mtarc1<br>Fetub<br>Vtn<br>Mst1<br>Grb7<br>Cfi<br>Cbs<br>Orm2<br>Reep6<br>Aox3<br>Qprt<br>Pglyrp2<br>Rtn4rl2<br>Plppr3<br>Exoc3l<br>Zap70<br>Rcor2

Groups	No. Of genes	Gene symbol
Mlkl$^{-/-}$ d3 PPE : Ripk1$^{D138N/D138N}$ d3 PPE	11	C4bp<br>Abcg5<br>Klkb1<br>Urah<br>Abcc2<br>C8g<br>4931406B18Rik<br>Gm11346<br>Il1a<br>Csta2<br>Il23a
Mlkl$^{-/-}$ d3 PPE : Mlkl$^{A/A}$ d3 PPE	2	Cyp4a10<br>Lrmp

Supplemental Figure S6: (**A**) Enrichment analysis for downregulated genes represented as top 10 GO terms and KEGG pathways as dot plots from *Mlkl$^{-/-}$*-d3-PPE, *Mlkl^{AA}*-d3-PPE, and *Ripk1$^{D138N/D138N}$*-d3-PPE animals. The size of the dots represent the number of genes in the significant DE gene list associated with the GO term and the color of the dots represent the P-adjusted values. (**B**) List of genes commonly downregulated between the indicated groups.

A

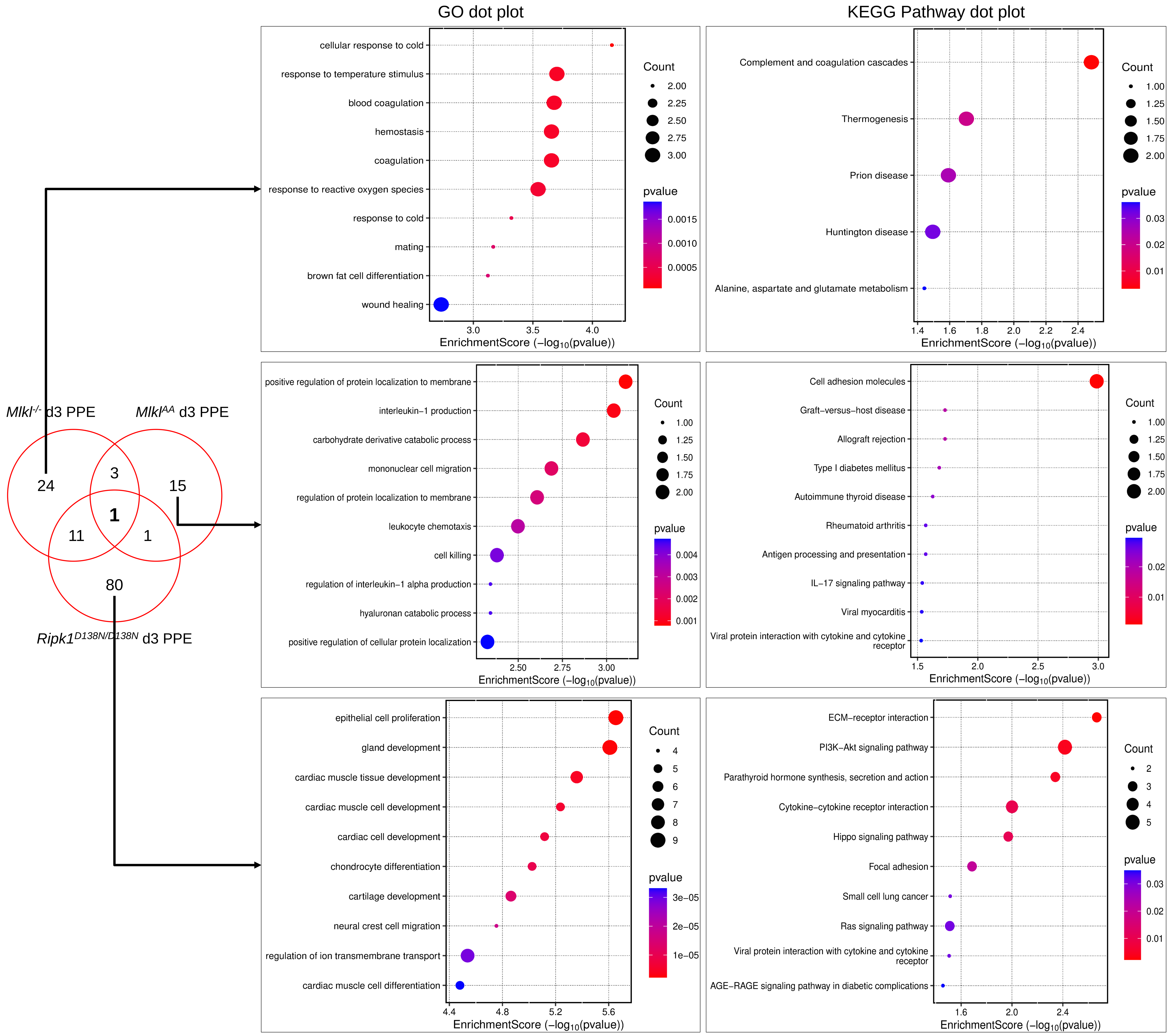

B

Groups	No. Of genes	Gene symbol
Mlkl$^{-/-}$ d3 PPE : Ripk1$^{D138N/D138N}$ d3 PPE	11	Cma1<br>Tpsb2<br>Mcpt4<br>Cytl1<br>Tnr<br>Cpa3<br>F2rl1<br>Mmrn1<br>Reln<br>Nkx3-1<br>Kcnj12
Mlkl$^{A/A}$ d3 PPE : Ripk1$^{D138N/D138N}$ d3 PPE	1	Gm15247
Mlkl$^{-/-}$ d3 PPE : Mlkl$^{A/A}$ d3 PPE	3	Gm15446<br>Tmem254b<br>Trpc6
Mlkl$^{-/-}$ d3 PPE : Mlkl$^{A/A}$ d3 PPE : Ripk1$^{D138N/D138N}$ d3 PPE	1	Mal

Supplemental Figure S7: (**A**) Enrichment analyses for upregulated genes represented as top 10 GO terms and KEGG pathways as dot plots from *Mlkl*$^{-/-}$-d3-PPE, *Mlkl*^{AA}-d3-PPE, and *Ripk1*$^{D138N/D138N}$-d3-PPE animals. The size of the dots represent the number of genes in the significant DE gene list associated with the GO term and the color of the dots represent the P-adjusted values. (**B**) List of gene commonly upregulated between the indicated groups.

A

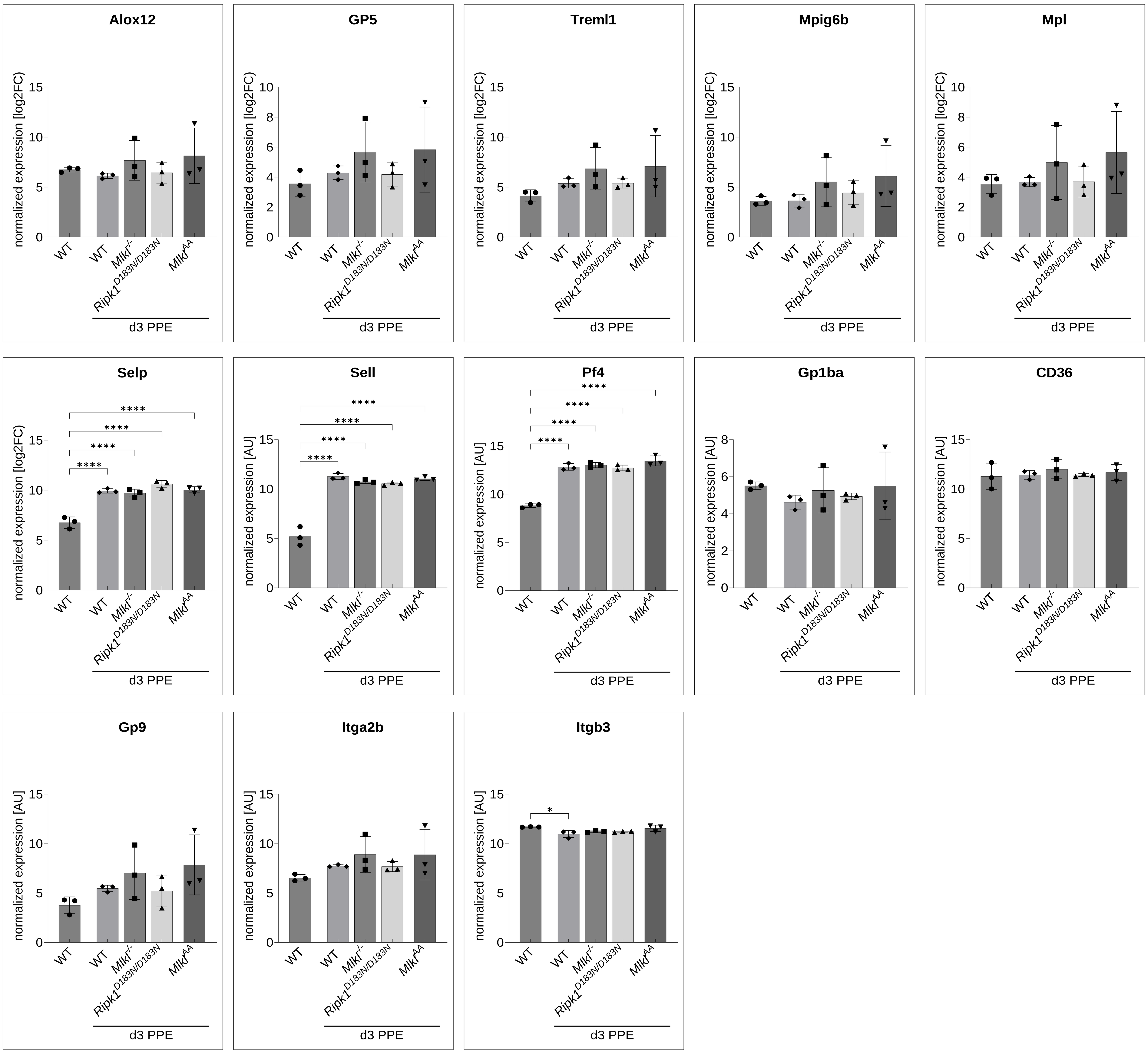

Supplemental Figure S8: (**A**) Genes associated with platelet activation and aggregation pathway with their log fold change values from WT-baseline and d3-PPE animals. Data are expressed as mean ± SD (n=3). Statistical significance was determined by Ordinary one-way ANOVA with Tukey's multiple comparisons test.

A

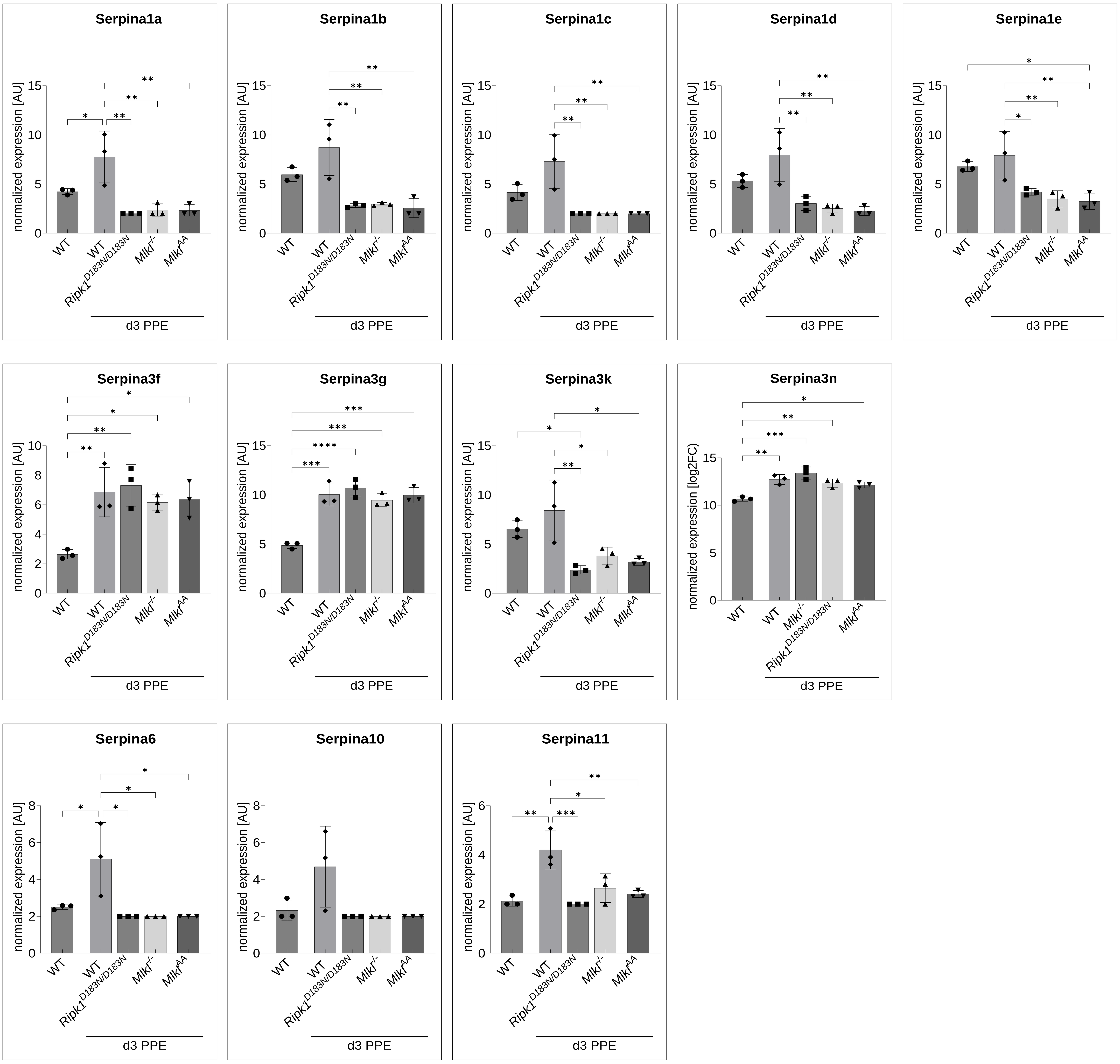

Supplemental Figure S9: (**A**) Genes associated with *Serpin* (serine proteases inhibitor) family with their log fold change values from WT-baseline and d3-PPE animals. Data are expressed as mean ± SD (n=3). Statistical significance was determined by Ordinary one-way ANOVA with Tukey’s multiple comparisons test.

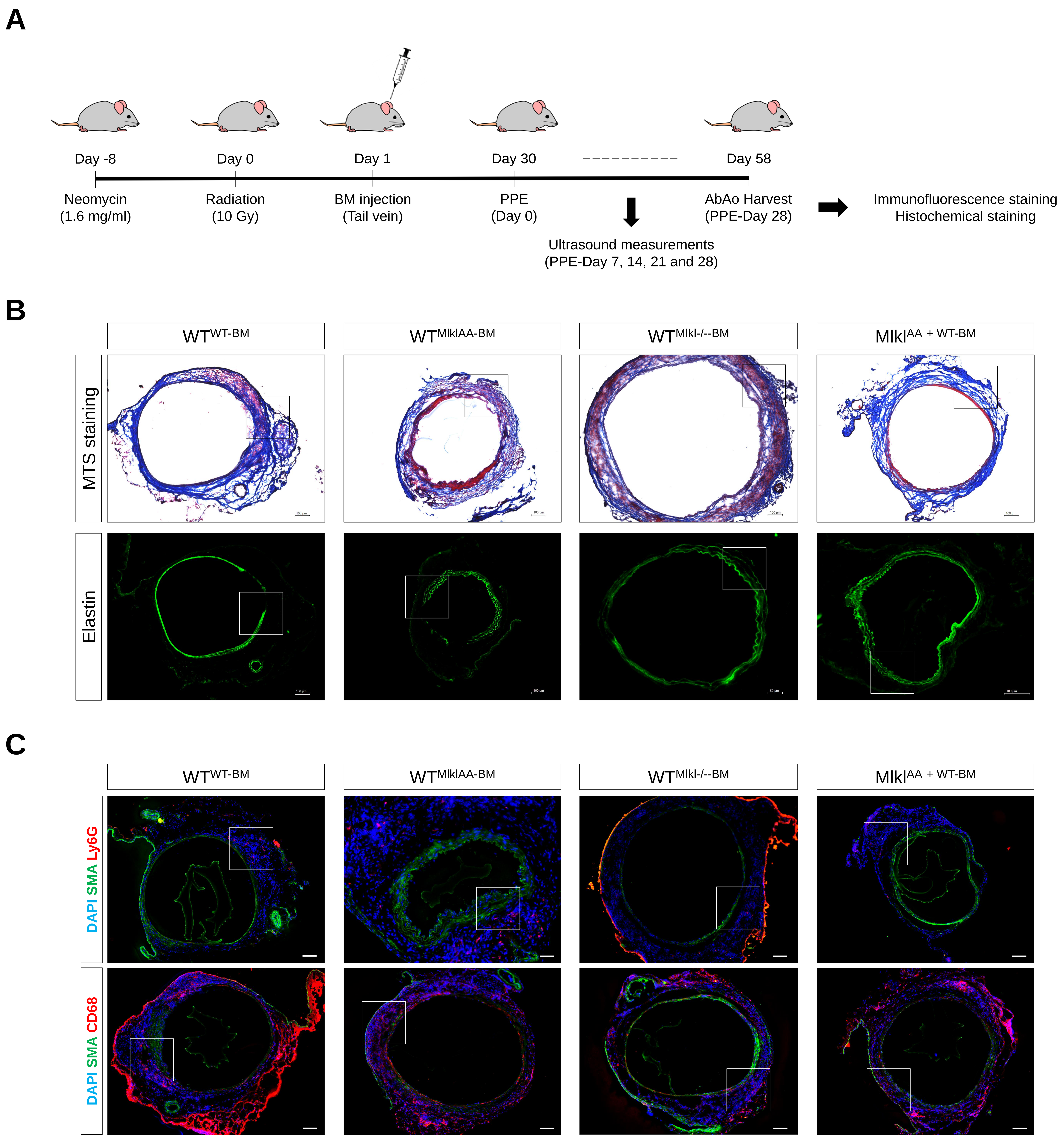

Supplemental Figure S10: (**A**) Experimental scheme of the bone marrow transplantation experiment. (**B**) Representative macroscopic images of complete abdominal aortic rings stained with Masson's Trichrome Staining (MTS) (top panel) and elastin autofluorescence (green; bottom panel). (**C**) Representative confocal images of complete abdominal aortic rings stained with aSMA (green) and Ly6G (red; top panel) or CD68 (red; bottom panel) (scale bars = 100 µm). Related to Figure 5D-G.

A

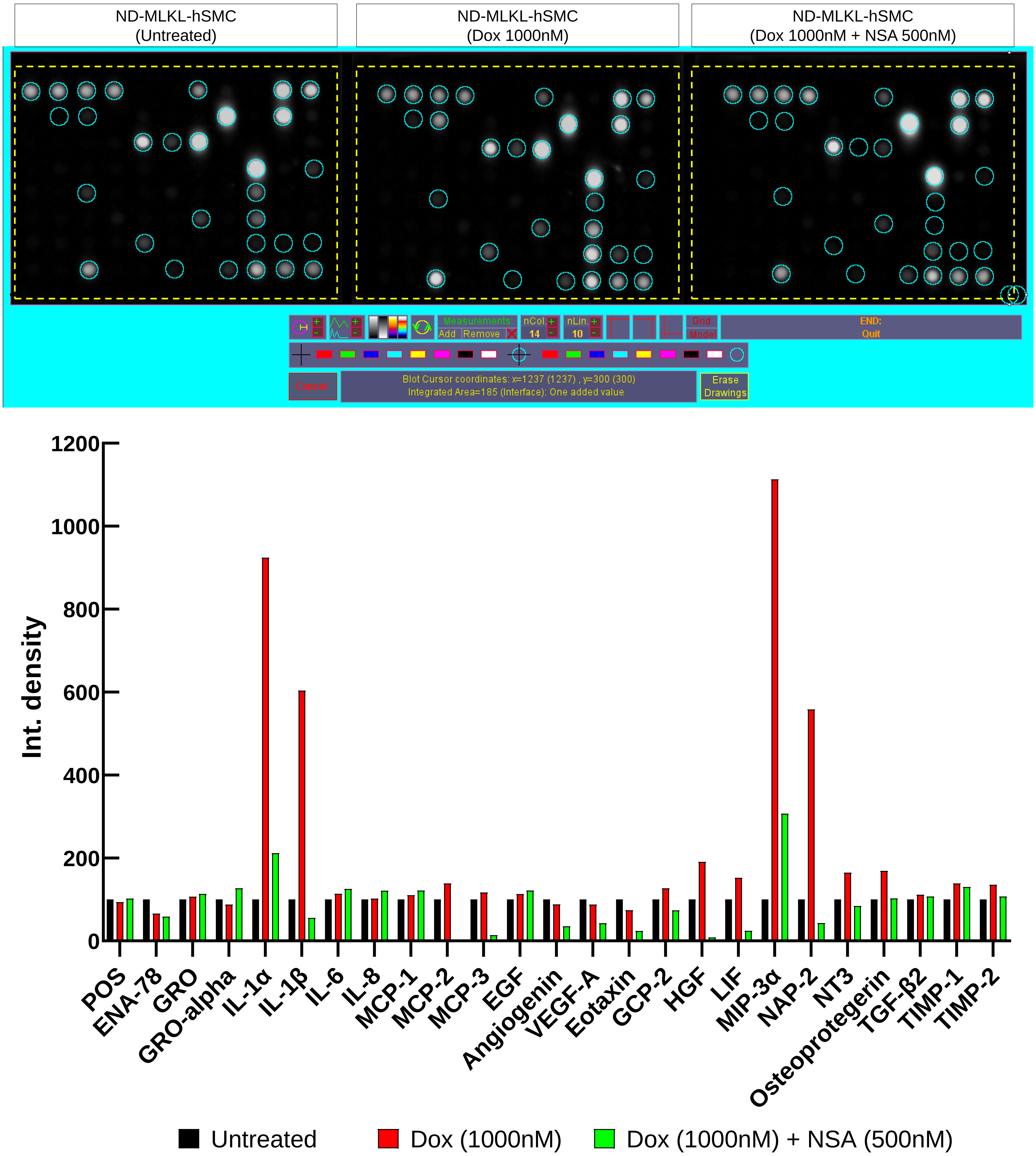

Supplemental Figure S11: (**A**) Quantification of cytokine array dot blots of untreated and induced ND-MLKL-hSMCs using ‘Protein Array Analysis’ tool in ImageJ. The integrated densities were calculated and normalized with respective integrated positive controls (POS) on each blot and then compared with untreated samples. Related to Figure 6E.

A

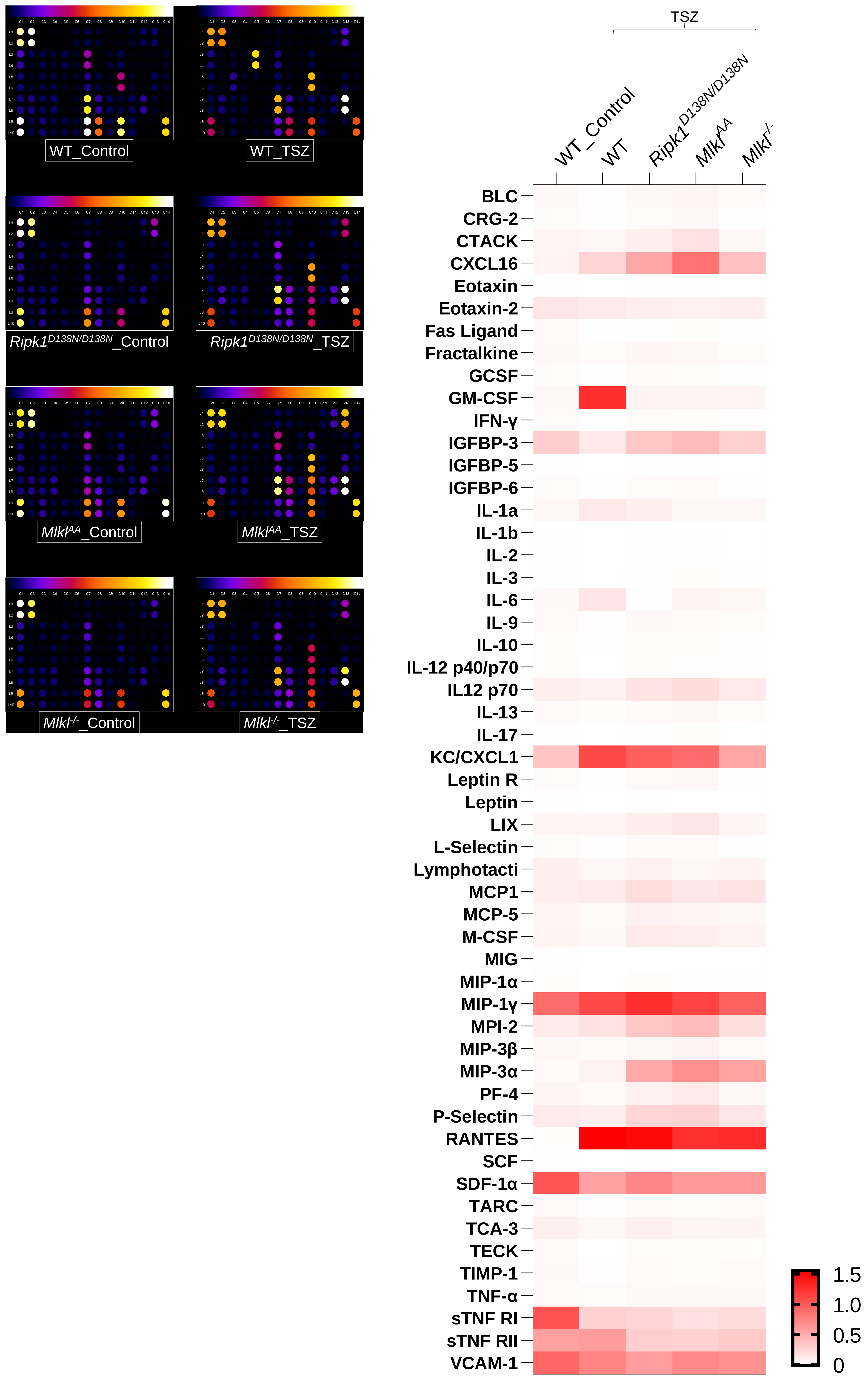

Supplemental Figure S12: (**A**) Representative dot blots of secretome antibody array analyses of the supernatant of control, and TSZ treated mouse primary aortic SMCs from WT, *Mlkl*$^{-/-}$, *Mlkl*^{AA} and *Ripk1*$^{D138N/D138N}$ animals (treatment: 48h; n=3). Related to Figure 6H.

A

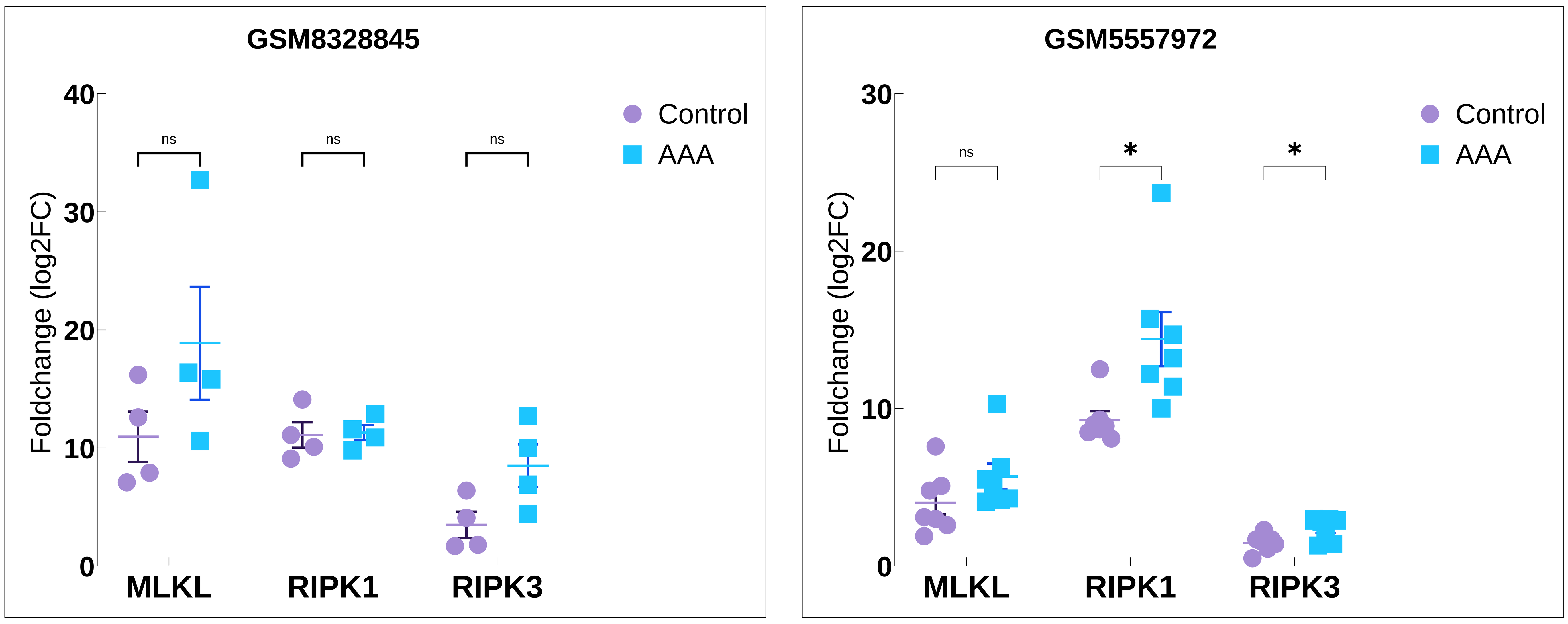

Supplemental Figure S13: (**A**) Expression analysis of necroptosis-related genes in human abdominal aortic aneurysm (AAA) samples. Levels of MLKL, RIPK1, and RIPK3 were assessed using publicly available transcriptomic datasets (GSM8328845, GSM55792).

Supplemental Video V1

Dox treated ND-MLKL-SMC cocultured with dPMN and live cell imaging was performed. The video shows typical necroptotic cell death with initial rounding of the cells followed by membrane rupture in the SMCs upon overexpression of ND-MLKL which was monitored indirectly via red mCherry fluorescence.

Supplemental Video V2

Dox treated ND-MLKL-SMC cocultured with dPMN and live cell imaging was performed. The video shows dPMN cells congregating around necroptotic SMC marked with red mCherry fluorescence.
